# Rigidification of the *Escherichia coli* cytoplasm by the human antimicrobial peptide LL-37 revealed by superresolution fluorescence microscopy

**DOI:** 10.1073/pnas.1814924116

**Published:** 2018-12-31

**Authors:** Yanyu Zhu, Sonisilpa Mohapatra, James C. Weisshaar

**Affiliations:** ^a^Department of Chemistry, University of Wisconsin–Madison, Madison, WI 53706

**Keywords:** antimicrobial peptide, LL-37, *E. coli*, nucleoid rigidification

## Abstract

Superresolution, single-particle tracking reveals effects of the cationic antimicrobial peptide LL-37 on the *Escherichia coli* cytoplasm. Seconds after LL-37 penetrates the cytoplasmic membrane, the chromosomal DNA becomes rigidified on a length scale of ∼30 nm, evidenced by the loss of jiggling motion of specific DNA markers. The diffusive motion of a subset of ribosomes is also frozen. The mean diffusion coefficients of the DNA-binding protein HU and the nonendogenous protein Kaede decrease twofold. Roughly 10^8^ LL-37 copies flood the cell (mean concentration ∼90 mM). Much of the LL-37 remains bound within the cell after extensive rinsing with fresh growth medium. Growth never recovers. The results suggest that the high concentration of adsorbed polycationic peptides forms a dense network of noncovalent, electrostatic linkages within the chromosomal DNA and among 70S-polysomes. The bacterial cytoplasm comprises a concentrated collection of biopolymers that are predominantly polyanionic (e.g., DNA, ribosomes, RNA, and most globular proteins). In normal cells, this provides a kind of electrostatic lubrication, enabling facile diffusion despite high biopolymer volume fraction. However, this same polyanionic nature renders the cytoplasm susceptible to massive adsorption of polycationic agents once penetration of the membranes occurs. If this phenomenon proves widespread across cationic agents and bacterial species, it will help explain why resistance to antimicrobial peptides develops only slowly. The results suggest two design criteria for polycationic peptides that efficiently kill gram-negative bacteria: facile penetration of the outer membrane and the ability to alter the cytoplasm by electrostatically linking double-stranded DNA and 70S-polysomes.

## Significance

Natural antimicrobial peptides (AMPs) that exhibit broad-spectrum antibacterial activity are often highly positively charged. Fluorescence microscopy shows that after permeabilization of *Escherichia coli* membranes by the cationic AMP LL-37 a massive influx of peptide freezes the diffusive motion of the chromosomal DNA and a subset of ribosomes. Both are highly negatively charged. Cells cannot recover growth. We suggest that LL-37 forms noncovalent, electrostatic linkages between DNA strands and among polyribosomes, rigidifying the entire cytoplasm. While the preponderance of polyanionic biopolymers in the cytoplasm facilitates diffusion in normal growth, this same characteristic renders the bacterium highly susceptible to attack by polycationic AMPs. The results help explain why bacteria develop resistance to AMPs very slowly and may inform the design of new antibacterial agents.

The era of multidrug-resistant bacterial infections necessitates discovery of new antibacterial treatments (1–3). Natural antimicrobial peptides (AMPs, also called host-defense peptides) comprise an ancient class of short polypeptides (typically <40 aa) that exhibit broad-spectrum antibacterial activity against both gram-negative and gram-positive bacteria (1). They may serve as templates for the design of new antibacterial agents. A large subclass of AMPs is highly cationic and forms amphipathic helices on binding to lipid bilayers (1, 4). While many cationic AMPs are known to permeabilize model lipid bilayers and real bacterial membranes (1), there is a growing appreciation that the influx of cationic AMPs after membrane permeabilization can impair a wide variety of bacterial mechanisms, including inhibition of transcription and DNA replication, of cytokinesis, of cell-wall biosynthesis, and of enzymatic activity and protein synthesis (1, 5). However, after decades of intensive study, it is fair to say that clear relationships between AMP structure and killing mechanisms have not emerged. A deeper understanding of the effects of natural AMPs on their bacterial targets may facilitate the effort to design new antibacterial agents.

Most mechanistic studies of the bactericidal effects of AMPs have focused on bulk, planktonic cultures. These bulk assays reveal a variety of specific biophysical and biochemical events, often with time resolution on the order of several minutes. For example, bulk methods can distinguish disruption of the outer membrane (OM) from disruption of the cytoplasmic membrane (CM) using fluorogenic dyes, measure real-time release of K^+^ from the cytoplasm, monitor dissipation of the proton motive force, and detect many additional effects (6, 7). Recent work has employed imaging methodologies such as transmission electron microscopy (TEM) (8), immunofluorescence, and soft X-ray tomography (9) to directly observe the effects of AMPs on single cells. Those studies necessarily involve fixation and permeabilization of the cells, and they are typically carried out at a single time point after addition of the AMP. A handful of studies glean spatial and temporal information from single-cell imaging of live bacterial cells, with fluorescence microscopy the most common tool (7, 10–14).

The only known human cathelicidin, LL-37, is a particularly well-studied amphipathic, cationic AMP. In addition to antibacterial effects, LL-37 exhibits antifungal and antiviral activity and plays an immunomodulatory role (15, 16). It is expressed in epithelial cells and in neutrophils and macrophages, where it is stored in granules as the inactive proprotein hCAP18 (15, 16). Once activated by infection or cell damage, such cells degranulate and release hCAP18 to the extracellular environment (16). The serine protease proteinase-3 cleaves hCAP18 to produce the active LL-37 form (16). It is believed that positively charged LL-37 selectively attacks bacterial cells but inflicts much less damage on host cells due to the highly anionic surface of both gram-negative and gram-positive species. The high concentration of sterols in the outer leaflet of host cell membranes may also help prevent LL-37 from inserting into host cell membranes (15, 17). Previous work has shown that at sufficiently high concentration LL-37 not only permeabilizes the *Escherichia coli* OM and CM (11, 15, 16, 18, 19) but also induces oxidative stress (13).

Here we extend single-cell imaging methods to include superresolution, single-particle localization (20–22) and tracking (23) of several cytoplasmic components of live *E. coli* before and after permeabilization of the CM by LL-37. In particular, we locate and track DNA loci, ribosomes, the nonendogenous globular protein Kaede, and the DNA-binding protein HU. This enables us to correlate in real time the motion of each type of particle, the halting of cell growth, and the permeabilization of the CM.

Shortly after LL-37 has permeabilized the CM of *E. coli*, the jiggling motion of the DNA loci halts completely; the chromosome has evidently rigidified. The diffusive motion of a subset of ribosomes, most likely polysomes, also halts completely on a length scale of ∼30 nm. The nucleoid volume expands somewhat and the degree of DNA–ribosome mixing increases. The average diffusion coefficient of the tetrameric exogenous protein Kaede and the dimeric DNA-binding protein HU decreases by a factor of two. Once the OM and CM are permeabilized, a remarkably large influx of LL-37 occurs, leading to an average LL-37 concentration inside the cell of ∼90 mM (∼10^8^ LL-37 copies per cell). We suggest that strong binding between polycationic LL-37 and polyanionic chromosomal DNA and ribosomes has produced a dense network of pseudo-cross-links that rigidifies the cytoplasm and inhibits proper movement of DNA, of ribosomes, and of globular proteins. Even after rinsing the permeabilized cells for 80 min with fresh, aerated medium lacking LL-37 the high intracellular density of LL-37 persists and growth does not resume. This live-cell study corroborates and extends a recent fixed-cell study from the Barron laboratory that used TEM and X-ray tomography to demonstrate agglomeration of ribosomes after treatment with LL-37 and a variety of cationic peptoids (9).

The vast majority of the biopolymers within the *E. coli* cytoplasm are polyanionic in nature (DNA, ribosomes, mRNA, tRNA, and the preponderance of globular proteins). It seems plausible that the resulting short-range repulsion between biopolymers provides a sort of “electrostatic lubrication” that enables reasonably facile diffusion of essential components, despite the high overall biopolymer density (24, 25). However, the present work suggests that the same polyanionic nature of the *E. coli* cytoplasm renders it highly attractive to polycationic AMPs and susceptible to a kind of electrostatic jamming action once the cationic peptide has permeabilized the bacterial membranes. If this phenomenon proves widespread across cationic AMPs and across bacterial species, it will help explain why bacteria develop resistance to cationic AMPs only very slowly.

## Results

### Freezing of Chromosomal Loci Movement When LL-37 Enters the Cytoplasm.

First we tracked the DNA locus *Right 2*, labeled by the fusion protein ParB-GFP (strain JCW154; *SI Appendix*, Table S1) (26, 27). The labeled ParB-GFP protein polymerizes specifically at a *parS* site engineered into the chromosome near the locus *Right 2*, forming bright puncta that can be tracked for some 300 frames with exposure time of 50 ms per frame without extensive photobleaching. In the first set of movies, we interleave three types of images with overall cycle time of 12 s per frame (Movie S1). In each cycle we collect images from phase contrast (enabling measurement of tip-to-tip cell length vs. time and defining the cell outline), from green fluorescence due to ParB-GFP foci, and from red fluorescence due to Sytox Orange (which fluoresces only after accessing the cytoplasm and binding to the chromosomal DNA). This enables us to monitor cell growth, the jiggling motion of the ParB-GFP puncta, and the onset of permeabilization of the CM by LL-37 over a period of 60 min. At time *t* = 0, we initiate flow of 4 µM of LL-37 [1× the 6-h minimum inhibitory concentration (MIC)] in warm, aerated EZRDM through the microfluidic chamber containing plated *E. coli* cells. The flow includes 5 nM of Sytox Orange.

**Fig. 1.**
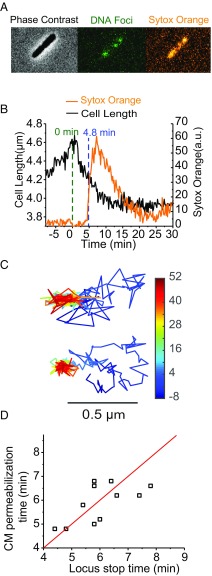
(*A*) Examples of phase contrast and two-color fluorescence images of a representative cell. (*Left*) Phase contrast monitors cell length vs. time. (*Center*) Green channel monitors ParB-GFP fluorescence to detect local DNA motion. (*Right*) Red channel monitors Sytox Orange fluorescence to detect CM permeabilization. (*B*) Cell length and Sytox Orange intensity vs. time for a single cell before and after injection of 4 µM LL-37 at *t* = 0 (green dashed line). Blue dashed line at *t* = 4.8 min marks the time when DNA loci motion halts, as judged by the trajectory in *C* at the top. (*C*) Time-lapse trajectories of two DNA loci over 1 h at 12 s per frame. Time in minutes is color-coded as shown. (*D*) Scatter plot of the time of CM permeabilization (from onset of Sytox Orange fluorescence) vs. the time when each DNA locus stops moving (judged by eye from the trajectory). Red line is a plot of *y* = *x*.

Typical phase contrast, ParB-GFP, and Sytox Orange images are shown in Fig. 1*A* for a representative cell. Cell growth, as inferred from cell length vs. time, halts within 2 min of addition of LL-37 (Fig. 1*B* and Movie S1). Cell length then gradually shrinks as LL-37 gains access to the periplasmic space. In earlier work (12), we attributed the gradual shrinkage to a stiffening of the peptidoglycan layer as LL-37 progressively binds to the anionic peptido cross-links. At *t* = 5 min, the CM is permeabilized and Sytox Orange rapidly gains access to the cytoplasmic space. We showed previously that LL-37 enters the cytoplasm at the same moment (11). Fifteen minutes after treatment with LL-37, the Sytox Orange observations show that essentially all cells have had their CM permeabilized (*SI Appendix*, Fig. S1). Accordingly, we designate cells observed at *t* > 15 min after LL-37 injection as “LL-37–treated cells.”

Within 1 min of permeabilization of the CM, the motion of the DNA foci changes abruptly. Before permeabilization, the motion is subdiffusive; the puncta mostly jiggle in place, typically sampling a length scale of ∼0.5 µm on a timescale of 5 min. After permeabilization, movement of the loci completely freezes within the resolution of the measurements. Two example trajectories are shown in Fig. 1*C*, with the progression of time coded in color. Additional trajectories are shown in *SI Appendix*, Fig. S2. We judge the time of freezing of the motion by eye as the first time of entry of the locus into the compact “trap,” after which it never leaves. Fig. 1*D* shows a plot of the time of CM permeabilization vs. the time at which DNA locus motion freezes. Different cells exhibit CM permeabilization at different times, ranging from about 4–10 min after LL-37 addition, indicative of heterogeneity across cells. The halting of DNA loci movement occurs at the same time within ±1 min.

**Fig. 2.**
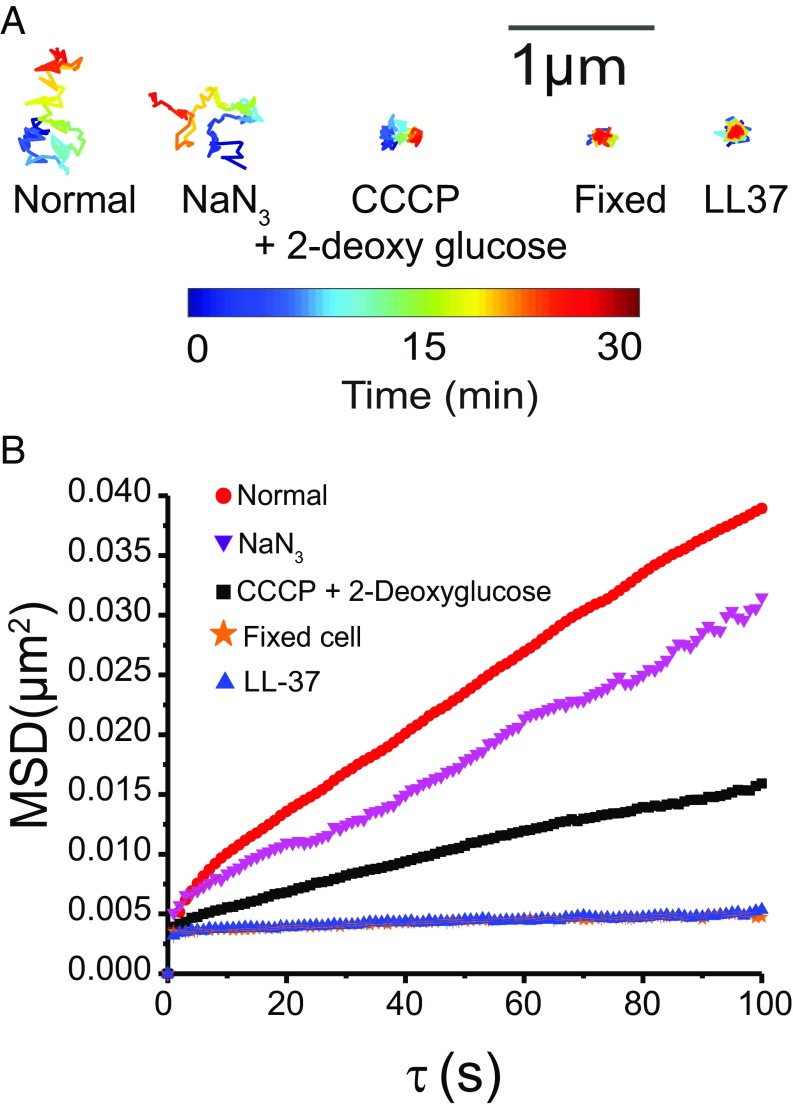
(*A*) Representative 30-min trajectories at 12 s per frame of DNA loci under treatments shown. The mean radius of gyration <*R*_*g*_> for each treatment is provided in *SI Appendix*, Table S2. (*B*) MSD vs. lag time for DNA loci under various treatments, obtained from movies taken at 1 s per camera frame for 600 frames. The apparent diffusion coefficient *D*_*app*_ is obtained from a linear fit to the first 10 points (*SI Appendix*, Table S2). Trend in *D*_*app*_ matches the qualitative trend in *A*. The numerical results are *D*_*app*_ = (2.0 ± 0.2) × 10^−4^ μm^2^⋅s^−1^ for normal growth, (9.1 ± 0.9) × 10^−5^ μm^2^⋅s^−1^ after NaN_3_ treatment, (4.6 ± 0.2) × 10^−5^ μm^2^⋅s^−1^ after CCCP treatment, (7.6 ± 2.1) × 10^−6^ μm^2^⋅s^−1^ for fixed cells, and (6.6 ± 2.4) × 10^−6^ μm^2^⋅s^−1^ after LL-37 treatment. The *R*^2^ values for the linear fitting are 0.968, 0.935, 0.983, 0.511, and 0.818, respectively.

Next we compare the movement of the DNA loci in five conditions: normally growing cells, cells after treatment with NaN_3_, cells after treatment with carbonyl cyanide *m*-chlorophenylhydrazone (CCCP) + 2-deoxy-glucose, which we call “CCCP treatment,” cells after fixation with formaldehyde, and cells after CM permeabilization by LL-37 (at *t* > 15 min). After 5 min of treatment with 15 mM NaN_3_, the pool of cytoplasmic ATP has been partially depleted. NaN_3_ inhibits ATP synthesis via the electron transport chain by blocking the action of cytochrome oxidase (28). Cells can still produce some ATP through glycolysis (28, 29). After 10 min of treatment with 200 µM CCCP + 1 mM 2-deoxyglucose, the pool of cytoplasmic ATP has been depleted completely. The ionophore CCCP eliminates ATP production by the ATP synthetase through dissipation of the proton motive force (30). Import of 2-deoxyglucose eliminates production of ATP by glycolysis (31). As judged qualitatively by the representative 30-min trajectories in Fig. 2*A*, NaN_3_ treatment reduces long-term loci movement only slightly. CCCP treatment reduces movement to a greater extent. LL-37 treatment reduces loci movement to a degree comparable to formaldehyde fixation. The corresponding average radius of gyration across trajectories for each condition shows the same trend (*SI Appendix*, Table S2).

We also carried out DNA loci tracking experiments at a faster rate of 1 frame per s. The corresponding mean-square displacement plots MSD(τ) on a 100-s timescale are shown in Fig. 2*B*. On that timescale, subdiffusion (negative curvature) is evident in all cases. The MSD plots after LL-37 treatment and after fixation are essentially indistinguishable. In general, the motion of the DNA loci is a superposition of “jiggling in place” and displacement of the center of gravity of the jiggling motion over longer times. Here we seek a simple, quantitative way to compare the motion under different experimental treatments. Accordingly, we estimate an apparent diffusion coefficient *D*_*app*_ in each case from the slope of the best-fit line to the first 10 experimental points. Results are summarized in *SI Appendix*, Table S2. Compared with normal growth conditions, NaN_3_ treatment decreases *D*_*app*_ by a factor of 2; CCCP treatment decreases *D*_*app*_ by a factor of 4. Fixation decreases *D*_*app*_ by a factor of 26. After CM permeabilization (*t* > 15 min), LL-37 decreases *D*_*app*_ by a factor of 30. The dynamic localization error estimated from the MSD intercepts is σ ∼ 30 nm. After fixation or LL-37 treatment, the slight apparent movement on a length scale of ∼30 nm over 100 s could be due primarily to sample drift during the measurements. Within the limits of our measurement accuracy, LL-37 has completely rigidified the chromosomal DNA.

### Slowing of Ribosome Diffusion.

**Fig. 3.**
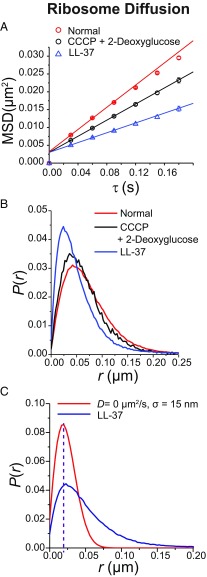
(*A*) Ribosome MSD vs. lag time τ from S2-mEos2 trajectories taken at 30 ms per frame in three different conditions as shown. The apparent diffusion coefficient *D*_*app*_ is obtained by linear fitting of the first three data points (*SI Appendix*, Table S2). The numerical results are (0.042 ± 0.001) μm^2^⋅s^−1^ for normal growth, (0.028 ± 0.001) μm^2^⋅s^−1^ after CCCP treatment, and (0.0180 ± 0.0005) μm^2^⋅s^−1^ after LL-37 treatment. *R*^2^ values are all 0.999; estimated localization errors are 34 nm, 32 nm, and 30 nm, respectively. (*B*) Ribosome single-step displacement distribution in same three conditions. (*C*) Ribosome single-step displacement distribution after LL-37 treatment (blue line) compared with a simulated distribution (red line) for a homogeneous population with *D* = 0 μm^2^/s and σ = 15 nm, with σ chosen so that the peak matches experiment. There is a slow ribosome subpopulation that is essentially motionless.

We also tracked ribosome diffusion as a function of time after LL-37 addition. These experiments use the strain MSG196, in which the chromosomal DNA is altered to append a photoconvertible mEos2 protein to the C terminus of the ribosomal protein S2 (*SI Appendix*, Table S1). In effect, we are tracking 30S ribosomal subunits, which may occur as either free 30S subunits or 30S subunits incorporated into translating 70S ribosomes, including 70S-polysomes (32, 33). The ribosome movies are acquired at 30 ms per frame (Movie S2). The mean trajectory length is 4.3 frames for normal conditions and 3.2 frames for LL-37–treated conditions. We analyze only those trajectories that last six steps or longer and truncate the longer trajectories at six steps. Galleries of trajectories before and after LL-37 treatment are presented in *SI Appendix*, Fig. S3. In Fig. 3, we compare the average ribosome diffusive motion in normal cells, in cells treated with CCCP, and in cells treated with LL-37 (at *t* > 15 min). Compared with normal cells, treatment with CCCP decreases the mean diffusion coefficient by a factor of 1.5. The mean apparent diffusion coefficient *D*_*app*_ of ribosomes in LL-37–treated cells has decreased by a factor of 2.3 (*SI Appendix*, Table S3). The same trend is shown by the distributions of single-step displacements *P*(*r*) (Fig. 3*B*). Treatment with CCCP narrows the distribution somewhat. Fifteen minutes after LL-37 addition, the ribosome distribution has narrowed substantially and the peak has shifted to much smaller values. In Fig. 3*C*, we show that the peak of the distribution matches that of a stationary population (*D* = 0) with measurement error σ = 15 nm. We show in *SI Appendix* that such a small measurement error is appropriate for slowly moving or stationary 30S copies (*SI Appendix*, Fig. S4). In addition, in *SI Appendix*, Fig. S1*A* we show that the ribosome diffusion slows on the same timescale as Sytox Orange entry into the cytoplasm. Below we will show that the slowest ribosomes concentrate in the three ribosome-rich regions after LL-37 treatment.

### Slowing of HU Diffusion.

**Fig. 4.**
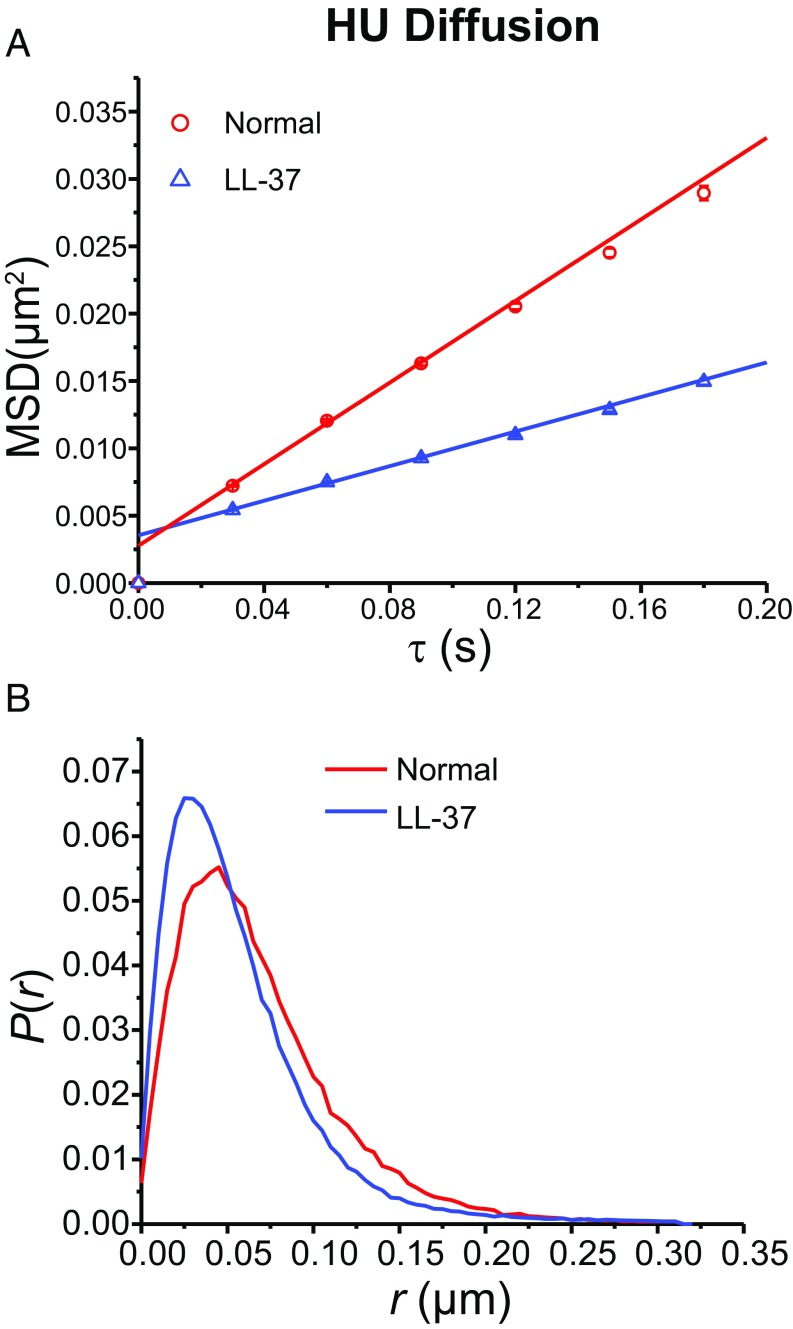
(*A*) HU-mEos2 MSD vs. lag time τ from movies taken at 30 ms per frame for normal and LL-37–treated cells (at *t* > 15 min). The apparent diffusion coefficient *D*_*app*_ is obtained by linear fitting of first three data points (*SI Appendix*, Table S3). The numerical results are (0.040 ± 0.001) μm^2^⋅s^−1^ for normal growth and (0.017 ± 0.001) μm^2^⋅s^−1^ after LL-37 treatment. *R*^2^ values are 0.999 and 0.998 respectively. Localization error is 32 nm in both normal condition and after LL-37 treatment. (*B*) HU single-step displacement distribution for normal and LL-37–treated cells.

HU dimer is a nucleoid-associated protein that binds nonspecifically to the chromosomal DNA (34–36). These experiments use strain JCW44, which contains a plasmid expressing HU labeled at the C terminus with the photoconvertible protein mEos2 (*SI Appendix*, Table S1). The mass of an HU–mEos2 dimer is 74 kDa. In earlier work we showed that expression of labeled HU from a plasmid does not alter the spatial distribution of DNA (37). Here we imaged HU at 30 ms per frame, both in normal cells and 15 min after treatment with LL-37 (Movie S3). Trajectories of six steps or longer were analyzed, and longer trajectories were truncated to six steps. The results are shown in Fig. 4. The MSD plots (Fig. 4*A*) show that for *t* > 15 min LL-37 treatment decreases the mean apparent HU diffusion coefficient by a factor of 2.3 (*SI Appendix*, Table S3). The distribution of single-step displacements (Fig. 4*B*) narrows considerably, and the peak shifts to smaller values.

### Slowing of Kaede Tetramer Diffusion.

**Fig. 5.**
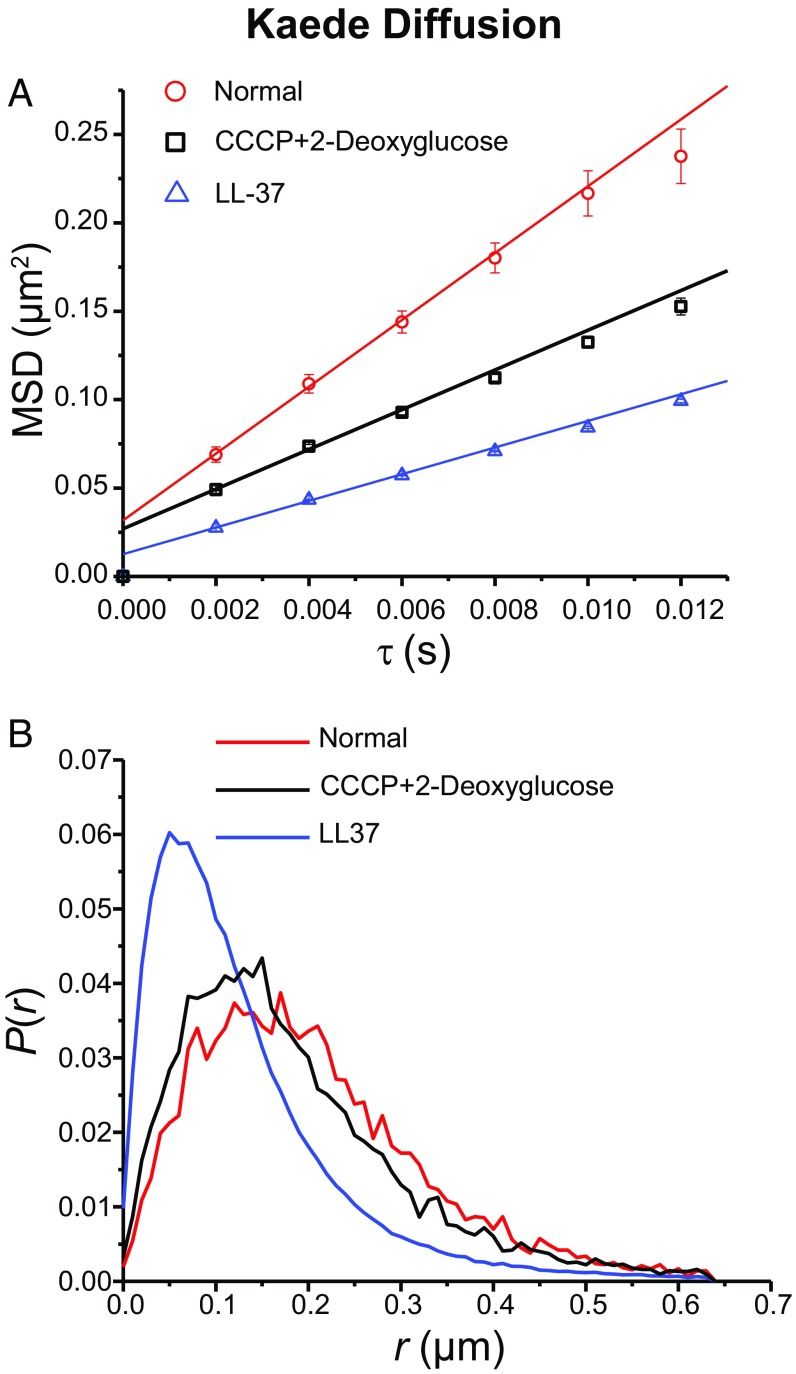
(*A*) Kaede MSD vs. lag time τ from movies taken at 2 ms per frame in three different conditions as shown. The apparent diffusion coefficient *D*_*app*_ is obtained by linear fitting of first three data points (*SI Appendix*, Table S3). The numerical results are (5.0 ± 0.2) μm^2^⋅s^−1^ for normal growth, (3.1 ± 0.2) μm^2^⋅s^−1^ after CCCP treatment, and (2.0 ± 0.1) μm^2^⋅s^−1^ after LL-37 treatment. *R*^2^ values are 0.999, 0.996, and 0.999, respectively. Localization errors are 100 nm, 90 nm, and 65 nm, respectively. (*B*) Kaede single-step displacement distribution in three different conditions.

Kaede is an exogenous, photoconvertible protein believed to exist as a tetramer of total mass 110 kDa (38). It neither binds to DNA nor does it have a biological function in *E. coli*. In these experiments, Kaede is expressed from a plasmid (strain JCW96; *SI Appendix*, Table S1). We imaged Kaede at 2 ms per frame in normal cells, in cells treated with CCCP + 2-deoxy-glucose, and 15 min after treatment of cells with LL-37 (Movie S4). Results are shown in Fig. 5. The MSD plots (Fig. 5*A*) show that CCCP treatment decreases the mean apparent diffusion coefficient by a factor of 1.6. LL-37 treatment decreases the mean apparent diffusion coefficient by a factor of 2.5 (*SI Appendix*, Table S3). The distributions of single-step displacements (Fig. 5*B*) show the same trend, with LL-37 treatment having a much larger effect than CCCP treatment. In *SI Appendix*, Fig. S1*B* we show that Kaede diffusion slows down on the same timescale as CM permeabilization to Sytox Orange occurs.

### Partial Alleviation of DNA–Ribosome Segregation upon LL-37 Treatment.

**Fig. 6.**
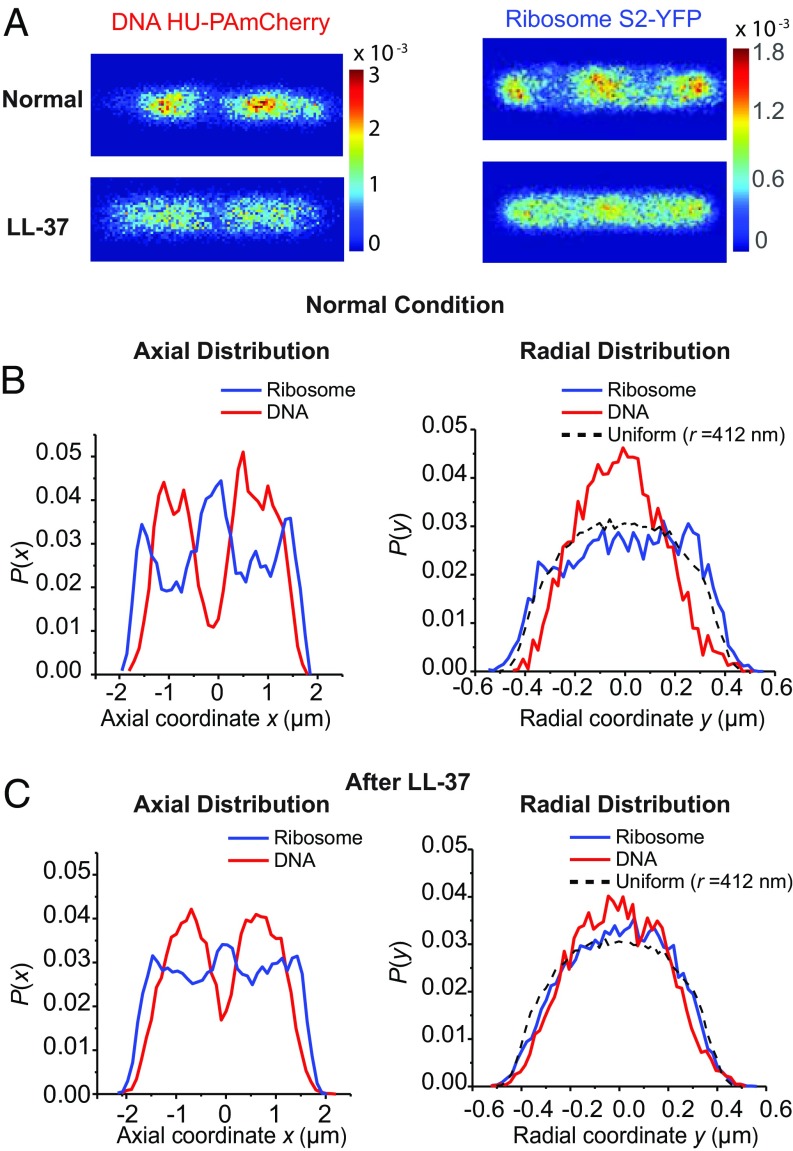
(*A*) Two-dimensional heat maps of DNA (HU-PAmcherry, *Left*) and ribosome (S2-YFP, *Right*) spatial distributions averaged across cells in normal growth (top row) and at *t* > 15 min after LL-37 treatment (bottom row). Only cells in the length range 4.0–4.3 μm were included. Pixel size is 30 × 30 nm. Color scale is in probability per pixel, so that the sum over all pixels equals 1 in each image. (*B*) Ribosome (blue) and DNA (red) axial distribution (*Left*) and radial distribution (*Right*) during normal growth. Averages over cells in the length range 4.0–4.3 μm. Model of a uniform radial distribution including localization error is shown as a dashed line for comparison. The radial distribution only includes the molecules in the nucleoid region (0.4 μm < |x|< 1.2 μm). (*C*) Ribosome (blue) and DNA (red) axial distribution (*Left*) and radial distribution (*Right*) at *t* > 15 after LL-37 treatment. Averages over cells in the length range 4.0–4.3 μm. Model of a uniform radial distribution including localization error is shown as a dashed line for comparison. The radial distribution only includes the molecules in the nucleoid region (0.4 μm < |x|< 1.2 μm).

Previous work (32, 33, 39, 40) showed that cells growing normally in EZRDM medium at 30 °C exhibit strong segregation of the chromosomal DNA from the ribosomes. For typical cells under these conditions, the DNA is distributed in two nucleoid lobes which interleave three “ribosome-rich regions” (Fig. 6). The ribosomes concentrate in the two endcaps, at the cell center, and in the thin annular region surrounding the nucleoid lobes. To measure both the DNA and ribosomal spatial distributions from the same cells we use strain SM6 (*SI Appendix*, Table S1), which expresses the ribosomal protein S2-YFP (green channel) from the chromosome and the construct HU-PAmCherry (red channel) from a plasmid. We recently showed that the distribution of HU is a good proxy for the overall distribution of chromosomal DNA, as judged by quantitative comparison with the staining pattern of the dye Sytox Orange (37). Single S2-YFP copies are imaged first in the green channel with an exposure of 30 ms per frame. We then imaged HU-PAmcherry in the red channel with an exposure of 30 ms per frame.

In Fig. 6*A*, we show 2D heat maps of the resulting superresolution spatial distributions of both HU and ribosomes in normal growth and at *t* > 15 min after LL-37 treatment. To minimize blurring of the spatial distributions, these are composite distributions taken from ∼50 cells whose length lies in the range 4.0–4.3 µm as determined from the tip-to-tip length in phase contrast images. Visual inspection suggests that LL-37 treatment has decreased the degree of DNA–ribosome segregation compared with normal growth. For a more quantitative view we project each 2D distribution onto the long cell axis (*x*) to form axial 1D distributions. The 1D radial distributions are obtained by projecting molecules in the nucleoid region onto the short cell axis (*y*). For normal growth (Fig. 6*B*), the DNA and ribosomes exhibit strong segregation, both axially and radially. In addition to the three axial ribosome-rich regions, ribosomes are also concentrated in the thin annular region surrounding the nucleoid lobes. After LL-37 treatment, the degree of segregation is diminished, both axially and radially (Fig. 6*C*). The axial distributions show three minor peaks for ribosomes and two substantial peaks for DNA, evidence of some residual segregation. The shoulders on the radial distribution of ribosomes are attenuated. The nucleoid has expanded, especially radially. The same distributions are replotted in *SI Appendix*, Fig. S5 in a way that clearly shows the changes induced by LL-37.

We can also quantify the degree of DNA–ribosome segregation in the 2D distributions of Fig. 6*A* using a modification of the Pearson correlation coefficient appropriate for the projection of 3D spatial distributions from spherocylinders into 2D images, as described in detail elsewhere (37, 41). We call the resulting modified Pearson correlation coefficient MPCC. The MPCC takes on the value +1 for the projection of two perfectly correlated images in 3D, –1 for the projection of two perfectly anticorrelated images in 3D, and 0 for the projection of two uncorrelated, random distributions in 3D. The results are MPCC = –0.31 in normal growth conditions and MPCC = –0.036 after LL-37 treatment. As before (37, 41), we test for statistical significance by comparison with simulations of randomly distributed 3D distributions in a spherocylinder of appropriate size with the same number of molecules and the same pixelation scheme as in the experiment. The value MPCC = –0.31 for normal cells is significantly different from 0; the probability that two random distributions would give a value at least that negative is *P* ∼ 10^−28^. The value MPCC = –0.036 after LL-37 treatment is only marginally significantly different from 0 (*P* ∼ 0.14). By this measure, LL-37 has destroyed much of the DNA–ribosome anticorrelation.

In *SI Appendix*, Fig. S3 we compare a gallery of single-ribosome trajectories for normal growth with a gallery of trajectories after LL-37 treatment. The latter are generally more compact and, indeed, LL-37 treatment appears to halt the motion of many ribosomal units. In addition, we can plot the location of the slowest and the fastest ribosome steps in normal growth and after LL-37 treatment. In *SI Appendix*, Fig. S6 we provide axial and radial distributions of each component. The slowest ribosome steps after LL-37 treatment exhibit three axial peaks, with the degree of modulation much like the distribution of all ribosomes in normal conditions. The fastest ribosome steps after LL-37 treatment are more homogeneously distributed. In normal growth, 70S-polysomes (slowly diffusing copies) are strongly segregated from DNA while free 30S subunits (rapidly diffusing copies) are not (37, 39). The distribution of the slowest copies after LL-37 treatment suggests that the copies whose motion is essentially frozen (Fig. 3*C*) tend to be 70S-polysomes, not free 30S subunits.

### LL-37 at Twice the MIC Disrupts ParB Foci.

We also studied the ParB-GFP foci at an increased LL-37 concentration of 8 µM, twice the MIC. Abrupt freezing of the foci motion occurred much as before. However, we also observed that some of the sharp, clear fluorescent puncta “dissolved” into wider, dimmer fluorescent regions. Example images are provided in *SI Appendix*, Fig. S7 and Movie S5. We speculate that LL-37 at this higher concentration can disrupt the binding of ParB-GFP to the chromosome (42, 43).

### Estimates of the Mean Number of LL-37 Copies Absorbed per Cell.

Quantitative interpretation of the diffusion results is aided by an estimate of how much LL-37 is absorbed per cell at the killing concentration of the peptide. Our method is similar to that used previously by the Wimley laboratory (44). For these experiments we use the mutant peptide LL-37 (F27W) to enhance absorption at 280 nm; the MIC of 4 μM remains unchanged (*SI Appendix*, Table S4). We first incubate various concentrations of LL-37 with various total cell counts (colony-forming units per mL) in PBS solution for 60 min in a test tube. Then we aliquot each sample into 96-well plates supplied with EZRDM, where they are incubated for 6 h. If the optical density does not increase over 6 h, then that concentration of LL-37 was large enough to kill the initial number of cells (*SI Appendix*, Fig. S8). This determines the minimum bulk LL-37 concentration required to kill a given number of cells and sets an upper bound on the amount of LL-37 absorbed per cell at the killing concentration.

Next we determine the absolute amount of LL-37 absorbed per cell by mixing a known number of cells with the minimum killing concentration of LL-37. After 60 min, the cells were removed by centrifugation. The supernatant was then analyzed for the total amount of LL-37 not absorbed by the cells, using HPLC and absorption at 280 nm. Given the amount of LL-37 not absorbed by the given number of cells and the amount of LL-37 originally present, subtraction yields an estimate of the number of LL-37 molecules absorbed per cell (*SI Appendix*, Fig. S9). For an initial LL-37 concentration of 20 μM, our best estimate is ∼2.1 × 10^8^ absorbed LL-37 per cell. Details are provided in *SI Appendix*.

The conditions in the experiments measuring LL-37 uptake are different from those in the microscopy experiments (*SI Appendix*). In the discussion to follow we take ∼10^8^ LL-37 copies absorbed per cell as a rough estimate of the LL-37 uptake at killing concentration on the timescale of the diffusion measurements. This is comparable to estimates for uptake of other AMPs by *E. coli* at cell-killing dosages (44, 45). Given a typical cell volume of *V*_*cell*_ ∼ 1.9 μm^3^ under these growth conditions (37), the estimated mean LL-37 concentration within a cell after CM permeabilization becomes ∼90 mM, a very large number compared with the bulk concentration of 20 μM.

### Recovery Experiments and Persistence of LL-37 Binding After OM Permeabilization.

Next we tested whether cells could recover after LL-37 treatment and whether the LL-37 that is bound within the cell could be rinsed away. These measurements use rhodamine-labeled LL-37 to enable us to use fluorescence to monitor the peptide bound within the cell. First, EZRDM containing Rh-LL-37 at 4 μM (1× the MIC) was flowed across plated cells for 20 min, a sufficient time to cause all of the plated cells to suffer the symptoms described above. The flow was then switched to fresh, aerated EZRDM (without peptide) for a subsequent period of 80 min, the recovery period. Throughout the incubation period and the recovery period, phase contrast and red fluorescence images of Rh-LL-37 were obtained at 2-min intervals, a period chosen to prevent significant photobleaching. Results are shown in *SI Appendix*, Fig. S10. As before, for all cells monitored growth halted within minutes of LL-37 introduction. Red, intracellular fluorescence from Rh-LL-37 increased upon membrane permeabilization, reached a plateau, and then increased gradually over the subsequent 80-min recovery period. The gradual increase is likely due to partial dequenching of intracellular fluorescence as some of the Rh-LL-37 is rinsed away. On the 80-min timescale, there was no evidence of recovery of growth from phase contrast imaging. Binding of LL-37 within the cell seems remarkably persistent over time.

In two follow-up experiments, we tested for the possibility that much of the Rh-LL-37 is retained within the cells because one or both of the membranes has resealed. In the first experiment, we flowed unlabeled LL-37 across unlabeled cells for 1 h, after which the flow was switched to include Sytox Orange plus LL-37. Fluorescence from DNA-bound Sytox Orange rose immediately (*SI Appendix*, Fig. S11*A*). The second experiment more closely mimics the rinsing experiment. We flowed 1× MIC LL-37 for 20 min, followed by rinsing with fresh medium for 60 min (no LL-37), followed by rinsing with fresh medium plus Sytox Orange (no LL-37). Again the Sytox Orange fluorescence rose immediately (*SI Appendix*, Fig. S11*B*). Evidently both membranes remain permeable long after the initial attack by LL-37. However, in the rinsing experiments of *SI Appendix*, Fig. S10 much of the Rh-LL-37 was retained during prolonged rinsing. We discuss this result further below.

## Discussion

There is a growing awareness that the damage inflicted on bacterial cells by AMPs goes well beyond permeabilization of their membranes (1, 5). In work related to the present study, the Barron laboratory recently carried out a TEM and X-ray tomography study of the structural effects of LL-37 and various cationic peptoids within the *E. coli* cytoplasm (9). The images reveal agglomeration of ribosomes within the cytoplasm.

Here we have used live-cell imaging to show that entry of some 10^8^ LL-37 copies per cell following permeabilization of the CM drastically decreases the diffusive motion of several key cytoplasmic components. The jiggling motion of the DNA locus *Right2* essentially freezes within 1 min of CM permeabilization (Figs. 1 and 2). The average diffusion coefficient of ribosomes decreases by about a factor of two, while the motion of a substantial subpopulation essentially freezes (Fig. 3). These frozen ribosomes concentrate in the three ribosome-rich regions, as do 70S-polysomes in normal growth conditions (*SI Appendix*, Fig. S6). This suggests that at least some of the 70S-polysomes are frozen in place, consistent with the recent X-ray tomography study of LL-37–treated cells (9). At the same time, the degree of DNA–ribosome segregation decreases compared with cells growing normally (Fig. 6). The diffusion of both the exogenous protein Kaede tetramer (110 kDa; Fig. 5) and the endogenous DNA-binding protein HU-mEos2 dimer (74 kDa; Fig. 4) also slows down, again by a factor of two on average. Fifteen minutes after LL-37 addition, many small, globular, non-DNA-binding proteins have been lost to the cell surround, as witness the complete loss of cytoplasmic GFP (11, 13). By inference, small metabolites and NTPs have been lost as well. We seek unifying concepts that explain these phenomena.

Earlier work showed that ATP depletion can substantially decrease the motion of DNA loci (29, 46) and large species such as plasmids and storage granules (47). The effect was attributed to loss of metabolic activity. In our experiments, CCCP treatment diminishes DNA jiggling motion, but substantial motion remains detectable. However, as shown in Fig. 2, the effects of LL-37 treatment (which also depletes ATP) are more extreme than those of CCCP treatment and comparable to the effects of fixation with formaldehyde. Whatever level of thermal motion remains, it has dropped below our detection limit of ∼30 nm over 100 s.

The estimate of ∼10^8^ LL-37 copies absorbed per cell after CM permeabilization corresponds to a concentration averaged over the entire cell volume (cell membranes, periplasm, and cytoplasm) of ∼90 mM. The cell has concentrated LL-37 by a factor of ∼5,000 compared with the original bulk concentration of 20 µM used in the uptake experiments. At neutral pH, LL-37 (sequence LLGDFFRKSKEKIGKEFKRIVQRIKDFLRNLVPRTES) includes 11 positively charged residues and five negatively charged residues, plus the positive N terminus and the negative C terminus (net +6). The massive influx of LL-37 introduces roughly 6 × 10^8^ net positive charges. This charge is presumably compensated by loss of small cations from the cell and gain of some accompanying small anions.

Next we consider the magnitude and plausibility of these numbers. First, can so many LL-37 copies even fit inside the cell? By approximating LL-37 as an α-helix of diameter 0.75 nm and length 37 residues × 0.15 nm per residue = 5.6 nm, we estimate the volume occupied by each LL-37 to be ∼2.5 × 10^−9^ μm^3^. Thus, 10^8^ LL-37 copies would occupy roughly 0.25 μm^3^, or about 10% of the overall original cell volume *V*_*cell*_ ∼ 1.9 μm^3^ (37). Second, what could drive the cell to bind so much positive charge? Images of Rh-LL-37 taken after CM permeabilization are smooth and uniform, indicating that LL-37 fills the entire volume of the cell, presumably binding significantly to cytoplasmic, periplasmic, OM, and CM components.

Most important for this study is the mechanism of LL-37 binding within the cytoplasm, where most of the biopolymers are polyanions (24). We suggest that much of the LL-37 uptake by the cytoplasm is driven by strong, electrostatic binding between the polycationic peptide and anionic biopolymers including chromosomal DNA, ribosomes, tRNA, and so on. Binding of so many positively charged LL-37 molecules within the cytoplasmic volume will displace a corresponding number of small cations, primarily K^+^, to the cell surround. The resulting increase in entropy provides an additional driving force for the binding (48).

At the growth rate studied, the ∼2.3 chromosomes per cell = 10.6 × 10^6^ bp carry ∼2.1 × 10^7^ negative phosphate charges, compensated by the high cytoplasmic K^+^ concentration (49). The ∼50,000 ribosomes (33), each with charge of –4,500, carry a combined ∼2.2 × 10^8^ negative charges. Only ∼50% of these charges are compensated by ribosomal proteins and structural Mg^2+^ cations (49). That leaves ∼1 × 10^8^ ribosomal charges compensated by K^+^. The ∼375,000 tRNA copies (occurring mostly as ternary complexes of mass ∼70 kDa, large enough to prevent escape) carry ∼80 phosphates each for a total of ∼3 × 10^7^ negative charges (50). These cytoplasmic species alone provide ∼2 × 10^8^ negative charges residing in cytoplasmic polyanionic species that are compensated by K^+^. This is sufficient negative charge to support the suggestion that much of the LL-37 within the cytoplasm is noncovalently (primarily electrostatically) bound to the chromosomal DNA and to the ribosomes within the cytoplasmic volume. Accordingly, in cells growing in minimal 3-(N-morpholino)propanesulfonic acid-buffered medium, the cytoplasmic K^+^ concentration is ∼200 mM (49). If the K^+^ concentration were the same in our faster-growing, larger cells, the cytoplasmic K^+^ copy number would be ∼2 × 10^8^, comparable to the estimated influx of positive charge due to LL-37 uptake. We conclude that the polyanionic–polycationic binding mechanism is at least plausible; see below for a quantitative estimate of the LL-37/DNA-binding constant.

It is important to recognize that the ParB-GFP foci we are tracking are massive (42). Their brightness suggests that a typical focus comprises ∼50–100 polymerized copies, with total mass ∼3–6 MDa. Each focus is bound to the chromosomal DNA polymer and embedded in nearby DNA polymeric strands. Because of the size of the ParB-GFP foci and the finite spatial resolution of the measurements, we can only assert that the chromosomal DNA is rigidified on a length scale of ∼30 nm. It remains possible that smaller probes would reveal more substantial motion on that length scale. However, because larger-scale motion of a compacted polymer is a composite of movements on shorter length scales, it seems likely that rigidification in fact occurs on significantly smaller length scales than 30 nm.

We suggest that the apparent freezing of the motion of the ParB foci arises from noncovalent (primarily electrostatic), “pseudo-cross-linking” of nearly contiguous DNA strands that envelop the ParB foci, with the DNA strands spanned by the ∼6-nm-long LL-37 peptide. Accordingly, a recent model of the *E. coli* nucleoid found that the average nearest distance between DNA strands is ∼6 nm (51). Meanwhile, analogous pseudo-cross-links may occur among 70S-polysomes concentrated in the three ribosome-rich regions. In this picture, each pseudo-cross-link involves electrostatic interactions between LL-37 and nearby polyanions. Although the pseudo-cross-links around the DNA locus probably form, release, and reform rapidly (discussed below), if their density is sufficiently high then single-particle tracking of DNA loci with ∼30 nm spatial resolution will detect little or no motion. Accordingly, an average LL-37 density of ∼10^8^ molecules per 2 μm^3^ corresponds to an average volume per peptide of 20 nm^3^, suggesting typical peptide-to-peptide spacings on the order of 3 nm. While the number of local binding events presumably fluctuates in time, the net effect will be continuous strong binding.

How strong is the binding? A number of previous studies qualitatively demonstrate binding of LL-37 to dsDNA in vitro and in vivo (9, 15, 52, 53). Record et al. (54) have carried out quantitative studies in vitro of the binding of polycationic peptides (often polylysines) to DNA as a function of monovalent cation concentration [M^+^]. The PBS buffer used in the uptake experiments has [M^+^] = 0.16 M. In *SI Appendix* we treat LL-37 as a *Z* = +6 peptide and apply Record’s equation to estimate that under the conditions of the uptake experiment,

*K*_*obs*_ = [LD]/[L][D] ∼20,000 M^−1^ for binding of LL-37 to the phosphate charges on dsDNA. Here L is the free peptide ligand of charge +*Z*, D represents a free, unblocked phosphate binding site along the DNA backbone, and LD represents the peptide bound to a phosphate site on DNA. Assuming each +6 peptide blocks *n* = 6 phosphate sites from additional ligand binding, we apply the McGhee–von Hippel binding isotherm to estimate how many peptides are bound within the nucleoid and the local concentration of DNA-bound peptide.

In our growth conditions, the average cell contains ∼2.2 chromosome equivalents = 10.1 × 10^6^ bp of DNA, providing *n* = 2 × 10^7^ individual phosphate binding sites. The parameters *n* = 6, *K*_*obs*_ = 20,000 M^−1^, and [L] = 4.2 μM from the uptake experiments then yield the estimate ν = 0.045 for the fractional occupancy at equilibrium. This is 27% of the maximum possible fractional occupancy, ν_max_ = 1/*n* = 0.166, so the coverage is fairly dense. There are an estimated ν*N* ∼ 9 × 10^5^ bound LL-37 copies within the nucleoid. The nucleoid contains ∼1/ν = 22 phosphate charges per bound LL-37 copy. The ∼9 × 10^5^ bound LL-37 within a 0.1-μm^3^ nucleoid volume (37) corresponds to a local LL-37 concentration of ∼15 mM. This very rough theoretical estimate is similar to the experimentally estimated average LL-37 concentration of ∼90 mM bound within the entire cell. The approximate agreement lends credence to the proposal that the large LL-37 uptake per cell arises from strong binding that is primarily electrostatic in nature. See *SI Appendix* for additional details, including an estimate of the timescale τ_bound_ ∼ 200 μs on which an LL-37 copy remains bound to DNA. The calculations suggest that the long-term binding of much of the LL-37 during rinsing arises from myriad dissociation and rebinding events within the nucleoid, which is dense with binding sites.

In normal growth conditions, DNA compaction and the segregation of DNA from translating 70S ribosomes (primarily 70S-polysomes) is strong. This has been attributed to several factors, including segregative phase separation arising from DNA–ribosome repulsion (55–57); excluded volume effects due to DNA compaction that render the empty pockets or “cages” inside the nucleoid uncomfortably small for a 70S ribosome (32, 39, 58, 59); configurational entropy of the DNA polymer, which causes it to avoid the cell boundaries, especially the endcaps (59); macromolecular crowding due to the plethora of globular proteins, which further compacts the DNA by depletion forces (60–63); supercoiling of the DNA (36, 60, 64, 65); and pairwise binding of distal DNA strands by nucleoid associated proteins such as H-NS (36, 55, 60, 66, 67).

After the LL-37 influx, the nucleoid expands somewhat and DNA–ribosome mixing becomes more extensive (Fig. 6 *B* and *C* and *SI Appendix*, Fig. S5). Decoration of the chromosomal DNA and of ribosomes with strongly bound, polycationic LL-37 would partially alleviate the repulsion between DNA and ribosomes. In addition, loss of small globular proteins to the cell surround may relieve crowding effects that help compact the DNA. Competitive binding by LL-37 may also displace positively charged DNA-binding proteins such as H-NS, Fis, and HU. Loss of metabolic activity may decrease supercoiling arising from transcription. All of these effects would tend to expand the nucleoid, enabling ribosomes to fit more readily into the cages within. However, long, highly charged polycations such as a polylysine 92-mer are known to drastically condense DNA in vitro in a process known as coacervation (60, 68). We suggest that LL-37 is insufficiently cationic (having both positive and negative charges interspersed with neutral residues) and too short to cause significant compaction by coacervation. In any case, apparently the decompaction forces induced by LL-37 outweigh the compaction forces. It would be interesting in this regard to study the effects of the highly cationic Gellman copolymers (69) on the degree of nucleoid compaction.

If the nucleoid expands, why then do the DNA-binding protein HU and the nonbinding, exogenous protein Kaede tetramer diffuse substantially more slowly after LL-37 treatment? For HU, which binds transiently and nonspecifically to DNA, we might expect competitive binding by LL-37 to produce a net enhancement of the mean diffusion coefficient due to an increase in the fraction of time HU copies are not bound to DNA. For both HU and Kaede, we might expect expansion of the DNA meshwork to enable faster 3D hopping from cage to cage within the nucleoid. Instead, the diffusion of both species slows down. A plausible explanation comes from a recent study by Chow and Skolnick (51), who modeled the nature of 3D diffusion of the transcription repressor protein LacI within the *E. coli* nucleoid. Their coarse-grained model found that thermal motion of the DNA strands themselves greatly facilitates hopping of LacI from one DNA cage within the nucleoid to an adjacent cage, a process termed “gated diffusion.” Similarly, we suggest that the rigidification of the DNA meshwork due to LL-37 binding slows the gated diffusion process, at least for proteins of the size of HU-mEos2 dimer (74 kDa) and Kaede tetramer (110 kDa). Diffusion of Kaede within the ribosome-rich regions may also be hindered by analogous rigidification due to pseudo-cross-linking of polysomes with each other. Meanwhile, the smaller protein GFP (27 kDa) readily escapes the rigidified cytoplasm (11, 13).

The results also lend some insight into the nature of the membrane disruptions induced by LL-37. Small globular proteins that do not bind DNA, exemplified by cytoplasmic GFP (27 kDa), are able to escape through the permeabilized CM on a timescale of seconds (11, 13). Apparently the permeabilization sites induced in the CM and OM by LL-37 are large enough to allow GFP to escape, but not so large as to allow Kaede tetramer, or the similar-sized HU-mEos2 dimer, to escape, at least not very rapidly. Our results do not speak to the long-standing issue of a toroidal pore vs. detergent-like permeabilization mechanism (1).

There are hints from our earlier work that other cationic AMPs have similar effects on diffusion and on DNA–ribosome mixing on entry into the *E. coli* cytoplasm. A widefield imaging study of the attack of Cecropin A (37 aa, +7 net charge) on *E. coli* showed essentially complete merging of the DNA and ribosome axial distributions at long times after CM permeabilization (70). The shorter, highly cationic, synthetic peptide CM15 (15 aa, +6 net charge) greatly slows the motion of DNA loci, much like LL-37 does (71). In addition, in a recent imaging study all but one of the cationic peptoids tested were found to agglomerate ribosomes (9).

## Conclusions

An inventory of the contents of the *E. coli* cytoplasm reveals a dense medium filled with polyanionic biopolymers (the chromosomal DNA, ribosomes, mRNA, tRNA, and most globular proteins) (24) that are stabilized and solvated by a host of small cations (mostly K^+^ and Mg^2+^). The biopolymer volume fraction in normal growth can be as large as ∼0.3 (72). There is little excess water beyond that required to provide several layers of solvation around the biopolymers. Poolman and coworkers (24) have plausibly suggested that short-range electrostatic repulsion among these polyanions provides a sort of lubrication that enables such a high total concentration of biopolymers to coexist and diffuse fairly rapidly within the cytoplasm.

However, our data strongly suggest that the same predominantly polyanionic character of the *E. coli* cytoplasm renders the bacterium highly susceptible to destructive, nonspecific binding interactions with polycationic peptides, including LL-37 and likely other cationic peptides as well. Once the OM and the CM have been permeabilized, the *E. coli* cytoplasm concentrates the cationic peptide LL-37 to a remarkable degree (∼90 mM inside the cell vs. 20 μM outside). Within the resolution of our single-particle measurements, the diffusive motion of chromosomal loci and of a subset of ribosomes (probably primarily 70S-polysomes) halts completely. We attribute this effect to a kind of pseudo-cross-linking of polyanionic biopolymers to each other, mediated by the polycationic peptide. Diffusion of midsize globular proteins slows down by roughly a factor of two. Rigidification of the nucleoid makes gated diffusion of globular proteins less facile. In addition, anionic patches on the surface of globular proteins may become partially neutralized by binding to the peptide, rendering the surrounding anionic biopolymer milieu “stickier.” Smaller globular proteins, small metabolites, and ATP are lost to the cell surround. LL-37 has transformed the cytoplasm from its normal dynamic state into a rigidified, gel-like state which inhibits proper motion of essential constituents.

It is difficult to imagine how a bacterial cell in such a condition could ever recover and grow again. Accordingly, our attempts to restore growth by flowing fresh medium across LL-37–treated cells were unsuccessful on an 80-min timescale. If such effects prove fairly general across cationic AMPs and across bacterial species, this would help explain why bacteria acquire immunity to AMPs only very slowly. Indeed, some of the most common resistance mechanisms developed by bacteria against cationic AMPs involve charge alterations of the outer surface or the CM that help diminish AMP binding and prevent membrane penetration from occurring in the first place (73).

For any given bacteria–AMP combination, it is generally not feasible to judge which particular AMP-induced effect, if any, is the proximate cause of cell death. In our experience studying *E. coli* interactions with a variety of cationic agents (7, 11–13, 69, 71), once the external AMP concentration is sufficient to permeabilize the OM, a cascade of subsequent events follows rapidly and irreversibly. This work and earlier work suggest two characteristics that may help determine cationic peptide potency against gram-negative bacteria: the ability to penetrate the OM and the ability to jam cytoplasmic diffusion by pseudo-cross-linking of the chromosomal DNA and of 70S ribosomes. Assays that test these specific properties could provide useful screens for new antimicrobial agents. Recent in vitro assays from the Barron laboratory used a gel retardation method to test binding to DNA and a ribosome flocculation OD measurement to test ribosome–ribosome cross-linking efficiency (9). The Wimley and Stella laboratories have devised methods to measure the absolute uptake of AMP per cell at cell-killing concentrations (44, 45). We recently demonstrated a HaloTag-based, single-cell fluorescence assay that detects passage of a small dye molecule, and presumably concomitant passage of a small peptide, across the OM of *E. coli* (74). Strong binding of AMPs to dsDNA and to ribosomes, as reported here, may enhance the potency of many antimicrobial agents.

## Methods

Overviews of each experimental method are provided in the main text. Additional details of the bacterial strains used, the LL-37 variants and their MICs, cell growth procedures, and preparation of samples for fluorescence microscopy studies are provided in *SI Appendix*. Microscopy procedures, data acquisition protocols, and procedures for analysis of single-particle trajectories are also provided. The procedure for estimation of LL-37 uptake per cell is modified from that of the Wimley laboratory (44); see *SI Appendix* for details.

## Supplementary Material

Supplementary File

Supplementary File

Supplementary File

Supplementary File

Supplementary File

Supplementary File
