# Supplementary material for: Rigidification of the Escherichia coli cytoplasm by the human antimicrobial peptide LL-37 revealed by superresolution fluorescence microscopy
Source: Proc Natl Acad Sci U S A. 2018 Dec 31;116(3):1017–26. doi: 10.1073/pnas.1814924116 (PMC6338858; doi:10.1073/pnas.1814924116)
Supplement: Supplementary File [file pnas.1814924116.sapp.pdf]

SUPPLEMENTAL INFORMATION

Table S1. Bacterial strains.

**Strain**	**Species Imaged**	**Strain Details**	**Background Strain**	**Expression Method**
JCW154	*Right2-parS* by ParB-GFP	Ref. (1)	MG1655	Plasmid
JCW44	HU–mEos2	Ref. (2)	MG1655	Plasmid
MSG196	Ribosome S2-mEos2	Ref. (3)	VH1000	Chromosome
SM6	Ribosome S2-YFP<br>HU-PAmcherry	Ref. (4)	VH1000	S2-YFP (Chromosome)<br>HU-PAmcherry (Plasmid)
JCW96	Kaede	Ref. (5)	VH1000	Plasmid

Table S2. Mean diffusion coefficient and radius of gyration from trajectories of the DNA locus *Right2* imaged at 1 frame/s or 12 s/frame as indicated.

**Cell Treatment**	$\boldsymbol{D_{app}}$ **(μm²/s)**[a] (1 s/frame)	$\boldsymbol{R^2}$ for D_{app}	**<$\boldsymbol{R_g}$> (μm)**[b] (1 s/frame)	**<$\boldsymbol{R_g}$> (μm)**[b] (12 s/frame)
Normal growth	$(2.0 \pm 0.2) \times 10^{-4}$	0.968	0.143 ± 0.005	0.30 ± 0.02
NaN_3	$(9.1 \pm 0.9) \times 10^{-5}$	0.935	0.138 ± 0.005	0.25 ± 0.02
CCCP+2-deoxyglucose	$(4.6 \pm 0.2) \times 10^{-5}$	0.983	0.080 ± 0.003	0.094 ± 0.008
LL-37 (t > 15 min)	$(6.6 \pm 2.4)\times 10^{-6}$	0.818	0.036 ± 0.001	0.089 ± 0.001
Fixed cells	$(7.6 \pm 2.1)\times 10^{-6}$	0.511	0.044 ± 0.002	0.083 ± 0.001

[a] D_{app} was obtained by linear fitting of first 10 points in MSD plot; ± values are fitting uncertainty. R^2 values after LL-37 treatment and for fixed cells are much less than one due to the small experimental slope compared with the noise level.

[b] **<$\boldsymbol{R_g}$>** is the mean of radius of gyration over all trajectories; ± values are ± 1 SD.

www.pnas.org/cgi/doi/10.1073/pnas.1814924115

Table S3. Mean diffusion coefficient of different species in normal growth and 15 min after onset of LL-37 treatment.

Species Tracked	D_{app} [a] ($\mu m^2/s$) Normal	R^2	D_{app} [a] ($\mu m^2/s$) after LL-37	R^2	Reduction Factor [b]	Frame time
DNA locus	$(2.0 \pm 0.2) \times 10^{-4}$	0.968	$(6.6 \pm 2.4) \times 10^{-6}$	0.818	0.033	1 s
HU-mEos2 dimer, 74 kDa	0.040 ± 0.001	0.999	0.017 ± 0.001	0.998	0.43	30 ms
Ribosome, 1340 kDa	0.042 ± 0.001	0.999	0.0180 ± 0.0005	0.999	0.43	30 ms
Kaede tetramer, 110 kDa	5.0 ± 0.2	0.999	2.0 ± 0.1	0.999	0.40	2 ms

[a] For DNA loci, D_{app} was obtained by linear fitting of first 10 points in MSD plot. For HU, ribosomes and Kaede, D_{app} was obtained by linear fitting of first 3 points in MSD plot. The ± values are the fitting uncertainty of the slope.

[b] Reduction factor is the ratio of apparent diffusion coefficient after LL-37 treatment to the apparent diffusion coefficient before treatment.

Table S4. Six-hour MIC values (μM) in EZRDM at 30°C for different LL-37 species.

**Peptide**	**LL-37**	**Rh-LL-37**	**LL-37 (F27W)**
**MIC (μM)**	4	4	4

Cell growth and preparation for imaging

Bulk cultures were grown in EZ rich, defined medium (EZRDM), which is a morpholinepropanesulfonic acid (MOPS)-buffered solution at pH = 7.4 supplemented with metal ions (M2130; Teknova), glucose (2 mg/mL), amino acids and vitamins (M2104; Teknova), nitrogenous bases (M2103; Teknova), 1.32 mM K_2HPO_4, and 76 mM NaCl. Cultures were grown from glycerol frozen stock to stationary phase overnight at 30°C. Subcultures were grown to exponential phase (OD = 0.2–0.6 at 600 nm) at 30°C before sampling for the microscopy experiments (6).

Strains used in the study are summarized in Table S1. Strains that express labeled species from a plasmid are grown with addition of 100 μg/mL ampicillin. When the cells reach mid-log phase, anhydrotetracycline was added to a final concentration of 45 nM to induce the expression of the labeled protein of interest. After 10 min of induction, the cells were centrifuged and resuspended in fresh growth media with 100 μg/mL ampicillin to remove the inducer. The cells were then incubated again in growth media for 15 min at 30°C to enable maturation of the labeled protein of interest prior to imaging.

For studying cells under ATP-depleting conditions, cells were treated with either 15 mM NaN_3 or with 200 µM carbonylcyanide-*m*-chlorophenylhydrazone (CCCP) plus 1 mM 2-deoxyglucose (7-12). These were added to the subcultures for 5 min and 10 min, respectively, prior to imaging. During the imaging, EZRDM was supplemented with each drug at the same concentration.

Two different imaging methodologies were employed, a flow chamber and a static chamber. Single-cell, time-lapse imaging experiments on the DNA locus *Right2* were carried out at 30°C in a PDMS-based microfluidics chamber consisting of a single rectilinear channel of uniform height of 50 μm, width of 6 mm and length of 11 mm. The total chamber volume is ~10 μL. After attachment of the PDMS chamber to the glass coverslip, 10 µL of 0.01% poly-L-lysine (molecular weight >150,000 Da) was flowed through the chamber and allowed to adsorb for 30 min. The chamber was then rinsed thoroughly with ultrapure water to remove excess poly-L-lysine. When the subcultures had grown to midlog phase, we flowed culture containing *E. coli* cells through the microfluidic chamber, followed by fresh, aerated, warmed EZRDM to wash away any unbound cells. The remaining cells are immobilized on the coverslip but grow normally. The PDMS ceiling of the microfluidics device is permeable to the ambient gases N_2

and O_2. The microfluidics chamber allows flowing of appropriate chemicals necessary for the experiment such as LL-37 (61302, Anaspec), Rh-LL-37(000-000-M33, Rockland Immunochemicals), or Sytox Orange (S11368, Thermo-Fisher Scientific), during imaging.

Single-molecule imaging of ribosomes (either S2-mEos2 or S2-YFP), HU dimer (labeled with either mEos2 or PAmcherry), and Kaede tetramer were carried out in a static chamber. First, ~150 µL of cell culture was placed within a CoverWell perfusion chamber gasket (Invitrogen) on a polylysine-coated, cleaned coverslip to fill the entire chamber volume. We allowed 2 min for the cells to adhere to the coverslip. The plated cells were then rinsed with the appropriate fresh, warmed, aerated media to wash away any non-adhered cells. For imaging of cells under normal growth conditions, the rinsing medium is EZRDM. Cells continue to grow normally for at least 30 min under these conditions. For LL-37 treated conditions, the rinsing medium is 4 µM of LL-37 (1X the 6-hr MIC) in EZRDM solution. The cells are maintained at 30°C throughout the imaging using an automatic temperature controller. For each species imaged, we also carried out a smaller set of single molecule experiments in the flow chamber. The results were essentially the same.

Minimum Inhibitory Concentration (MIC) Assay

The aerobic MIC value for the LL-37, Rh-LL37, and the mutated peptide LL-37 (F27W) (92875740001/PE4393, GenScript) were determined using the broth microdilution method as previously described (7). Two-fold serial dilutions of LL-37 in 1X EZRDM were performed in separate rows of a polystyrene 96-well plate, with each plate containing an inoculum of *E. coli* MG1655. The inoculum was a 1:20 dilution from a bulk culture at midlog phase (OD600 = 0.5) grown at 30°C. The plate was incubated at 30°C and shaken at 200 rpm in a Lab-Line Orbital Environ Shaker (Model 3527) for 6 hr. The MIC value was taken as the lowest concentration for

which no growth was discernible (<0.05 OD) after 6 hr. the MIC was the same 4 μM for all species (Table S4).

Microscopy

All imaging was performed on a Nikon Eclipse Ti inverted microscope (Nikon) with either an oil immersion 100×, 1.3 N.A. phase contrast objective or an oil immersion 100×, 1.45 N.A. phase contrast objective (CFI Plan Apo Lambda DM; Nikon Instrument). The images were further magnified 1.5× unless otherwise specified. Fast shutters (Uniblitz LS2; Vincent Associates) were used to synchronize illumination and image acquisition. Except for Kaede, images were recorded by a back-illuminated EMCCD camera with 16 μm x 16 μm pixels (either Andor iXon DV-897 or Andor iXon DV-887; Andor Technology). Each pixel corresponds to 105×105 nm^2 at the sample with an overall magnification of 150× unless otherwise specified. Kaede images were recorded by an Andor iXon 860 camera, with each pixel corresponding to 160×160 nm^2.

Studies of the motion of the DNA loci *Right2* labeled by ParB-GFP (strain JCW154; Figs. 1 and 2) were carried out in the flow chamber. ParB-GFP loci were imaged using 488 nm excitation (Coherent Sapphire laser), expanded to illuminate the field of view uniformly. The laser intensity was ~100 W/cm^2 at the sample plane. The emission filter was HQ525/50 (Chroma Technology). The loci could be tracked with good signal-to-noise for 300 camera frames.

Time-lapse movies of 60-min total duration were obtained with an exposure time of 50 ms each, with fluorescence and phase contrast images interleaved at 6-s intervals (12 s per complete cycle). Movies of 5 min total duration interleaved 50 ms fluorescence images with phase contrast images with the cycle time of 1 s per cycle. For dual color experiments imaging ParB-GFP (green channel) and Sytox Orange (red channel), μManager was used to obtain the data and

switch filters between frames using a LB10-NW filter wheel (Sutter). The time-lapse movies were obtained as 50-ms exposure time each, with green fluorescence (488 nm excitation), red fluorescence (561 nm excitation), and phase contrast images interleaved (12 s per complete cycle). To minimize spectral bleed-through in the two-color experiments, we utilized the narrower filters HQ510/20 for the green channel and HQ600/50M for the red channel. Laser intensities at the sample were typically ~100 W/cm^2 at 488 nm and ~2.5 W/cm^2 at 561 nm.

For superresolution co-imaging of single ribosomes and single HU copies (strain SM6 (4); Fig. 6), first the S2–YFP molecules were illuminated with the 514 nm laser (Coherent Sapphire) with a power density of ~2 kW/cm^2. Images were acquired only when the rate of return of molecules to the fluorescent state becomes small enough that at most 3-4 copies per camera frame are fluorescent in each cell, enabling single molecules to be distinguished from each other. After that, HU-PAmcherry molecules were photoactivated with 405 nm diode laser (CrystaLaser) and subsequently imaged with the 561 nm laser (Coherent Sapphire laser). The 405-nm power density at the sample was ~ 4-12 W/cm^2 to ensure that only 3 – 4 molecules of HU-PAmcherry are fluorescent per camera frame per cell. The power density of the 561 nm laser was ~2 kW/cm^2.We used dichroic ZT405-514-561rpc (Chroma). The fluorescence from S2-YFP and HU-PAmcherry was isolated using triple band pass filter ZET442/514/561m (Chroma). A 525 nm long pass filter (ET525lp, Chroma) was added to block the shorter wavelength band allowed by the triple band pass filter. To eliminate leakage of emission from YFP into the red channel after 561 nm excitation, we first imaged S2–YFP, which results in photobleaching of YFP molecules and enhances the contrast in subsequently obtained HU-PAmcherry images. HU- PAmcherry and Ribosome S2-YFP were imaged at a frame rate of 31.2 Hz, with an exposure time of 30 ms.

For single-particle tracking of HU-mEos2 (strain JCW 44, Fig. 4), ribosomes labeled by S2-mEos2 (strain MSG196, Fig 3), and Kaede (strain JCW 66, Fig 5), the fluorescent proteins were photoconverted using a 405 nm laser and subsequently imaged using a 561 nm excitation laser. The emission filter was HQ617/73 (bright line 617/73, Semrock). HU-mEos2 and ribosome S2-mEos2 were imaged at a frame rate of 31.2 Hz, with an exposure time of 30 ms. Kaede was imaged with a frame rate of 485.4 Hz, with an exposure time of 2 ms.

In all the experiments, the cells were imaged for <35 s per channel to ensure minimum cell damage by phototoxic effects of the laser and normal cell growth during the imaging period.

Data analysis

Images were analyzed using a MATLAB graphical user interface (GUI) developed in our lab (5). Images were smoothed and filtered to obtain a zero-based image. Bright spots were located with pixel-level accuracy by a peak finding algorithm that detects the local intensity maxima within an image. A user defined intensity threshold was used as the minimum brightness of a pixel arising from a single molecule. The threshold is carefully set by the user so that it will not be so high as to reject a real single molecule in the raw images or so low as to include background noise.

A modified MATLAB version of the tracking program written by Crocker and Grier (13) was used. As before (6), a centroid algorithm was used to locate the identified particles with subpixel resolution. Centroids of the bright spots were calculated from a 7 x 7 pixel square containing the entire bright spot, centered on the local maximum determined by the peak finding algorithm. The centroid positions from successive frames were connected to form a trajectory. For ribosomal and HU trajectories, the centroid positions are connected only if they lie within 3 px = 315 nm of each other. For Kaede, the centroid positions are connected only if they

lie within 4 px = 640 nm. For ribosomes and HU, each pixel is 105 nm x 105 nm, while for Kaede each pixel is 160 nm x 160 nm.

To generate the spatial distribution of molecules from several cells such as the one shown in Fig. 6, the camera based coordinates are reoriented so that the *x* axis and *y* axis correspond to the long and short cell axis, respectively. For identifying spatial localizations within the cell of choice, cell outlines generated by Microbe Tracker (14) from phase contrast images are used. Details are provided elsewhere (4).

In each experimental condition, the ensemble-averaged mean-square displacement was calculated as:

$$MSD(\tau) = \langle\left(\vec{R}(t+\tau) - \vec{R}(t)\right)^2\rangle = \frac{1}{N}\sum_i \frac{1}{m}\sum_m [\vec{R}_i(mt+\tau) - \vec{R}_i(mt)]^2. \qquad \text{(S1)}$$

Here *N* is the number of trajectories over which the ensemble average is taken. The index *m* runs from 1 to a specific value given the lag time τ, providing the time average of each trajectory. The final MSD is an average over the ensemble and over time. For DNA loci imaged with 1 s/frame time, *m* runs from 1 to 99. We used the linear fit of the first 10 points on the MSD plots to calculate an approximate apparent diffusion coefficient. For other particles including ribosomal S2-mEos2, HU-mEos2, and Kaede, *m* runs from 1 to 6 and we used the linear fit of the first 3 points on the MSD plots to calculate an approximate apparent diffusion coefficient via an average over thousands of trajectories (15).

Suppose the least-squares, best fit to the first 3 experimental points of a mean-square displacement plot is given by the equation MSD(τ) = $a + b\tau$, with *b* the slope and *a* the extrapolated intercept at lag time $\tau = 0$. Then Michalet (15) has shown that the most accurate mean diffusion coefficient is given by $D = b/4$ and the best estimate of the dynamic localization error is $\sigma = \frac{1}{2}(a + 4Dt_E/3)^{1/2}$, where t_E is the exposure time per camera frame. Mean diffusion

coefficients of different species in the various experiment of conditions are collected in Tables S2 and S3.

The radius of gyration R_g of each trajectory with m localizations was calculated using:

$$R_g = \sqrt{\frac{1}{m}\sum_{i=1}^{m}\left(\vec{R}(i) - \langle\vec{R}(i)\rangle\right)^2} \quad \text{(S2)}$$

where $\langle\vec{R}(i)\rangle = \frac{1}{m}\sum_{i=1}^{m}\vec{R}(i)$ is the average position of the particle in the trajectory. For a given set of experimental conditions, the mean radius of gyration (Table S2) is the average of R_g over all trajectories.

Modified Pearson Correlation Coefficient (MPCC)

The MPCC of two images **R** and **G** is evaluated as follows (16):

$$\text{MPCC} = \frac{\sum_{i=1}^{m}\sum_{j=1}^{n}(R_{ij}-\tilde{U}_{ij}^{R})(G_{ij}-\tilde{U}_{ij}^{G})}{\sqrt{\sum_{i=1}^{m}\sum_{j=1}^{n}(R_{ij}-\tilde{U}_{ij}^{R})^2}\sqrt{\sum_{i=1}^{m}\sum_{j=1}^{n}(G_{ij}-\tilde{U}_{ij}^{G})^2}}. \quad \text{(S3)}$$

Here m and n are the number of rows and columns in the image matrices **R** and **G**; there are m x n total pixels in each image. The R_{ij} and G_{ij} are the corresponding intensities of pixel ij in **R** and **G.** $\tilde{U}_{ij}^{R}$ and $\tilde{U}_{ij}^{G}$ denote the intensity of pixel ij in the 2D projection of a large set of molecules distributed randomly in a 3D spherocylinder. The total number of molecules in $\tilde{\mathbf{U}}^{R}$ and $\tilde{\mathbf{U}}^{G}$ has been scaled to be the same as the total number of molecules in **R** and **G**, respectively.

R and **G** are generated by pixelating the composite distribution of spatial localizations of HU-PAmcherry and S2-YFP molecules from cells in the chosen length bin to 50 nm. We simulated two random distributions of 100,000 molecules each, corresponding to the ribosome (green) and HU (red) channels, using a spherocylinder whose dimensions match those of the chosen cells. The resulting reference images are normalized to give $\tilde{\mathbf{U}}^{R}$ and $\tilde{\mathbf{U}}^{G}$, which have same number of molecules as imaged ribosomes and HU. The two simulated 3D random distributions, $\tilde{\mathbf{U}}^{R}$ and $\tilde{\mathbf{U}}^{G}$incorporated localization errors σ_{HU} and σ_{Ribo}, determined by the

intercepts of MSD plots for each species (Fig. 3A, 4A). The detailed procedure for generating $\tilde{\mathbf{U}}^R$ and $\tilde{\mathbf{U}}^G$ is described elsewhere (16).

Monte Carlo simulation of slow ribosome trajectories after LL-37 treatment

As detailed before (4, 5), we simulate the behavior of a homogeneous population (i.e., a population of molecules all with the same diffusion coefficient *D*) by calculating a large number of random walk trajectories. These simulations incorporate the appropriate dynamic localization error σ (from the intercepts of the MSD plot, as above) and confinement effects within a model spherocylinder that mimics the dimensions of a typical *E. coli* cell in our growth conditions (tip-to-tip cell length = 3.7 μm and cell diameter = 0.82 μm). These dimensions were chosen to fit the measured spatial distribution of Kaede molecules under the same growth conditions, under the assumption that Kaede distributes uniformly throughout the cytoplasm (4, 5).

For ribosomes after LL-37 treatment (Fig. 3), the peak of the single-step displacement distribution has shifted far to the left as compared with normal cells. The diffusion of a subpopulation of ribosomes has slowed down substantially. In order to understand the peak of the distribution, we ran a series of simulated trajectories with different combinations of diffusion coefficient *D* and localization error σ. The parameter combination that best explains the peak position uses *D* = 0 and σ = 15 nm, as shown in Fig. 3C. To test whether such a small localization error is reasonable for slow-moving ribosomes, we prepared the MSD plot for those six-step ribosome trajectories whose estimated diffusion coefficients fall in the lowest 15% of all trajectories. The results are shown in Fig. S4. After LL-37 treatment, the intercept corresponds to the localization error of σ_{slow} = 12 nm, comparable to the 15 nm used in the model single-step distribution that matches the experimental peak. This indicates that a substantial subset of

ribosomes are essentially frozen in place to the limits of our measurement accuracy after LL-37 treatment.

Number of LL-37 copies absorbed per cell

The method is based on that described previously by the Wimley lab to determine the average number of LL-37 copies absorbed per cell (17) for the lowest concentration of LL-37 that kills all the cells in the sample. We used the mutant peptide LL-37 (F27W) instead of normal LL-37 to increase the absorbance at 280 nm and enable the uptake measurements. LL-37 (F27W) has the same MIC as LL-37 (4 µM).

We first prepared combinations of different concentrations of LL-37 (F27W) and different initial cell counts to determine the number of peptide molecules needed to kill the bacteria at different cell counts. We grew cells to OD = 1.0 or higher in EZRDM, then centrifuged and resuspended the cells in 1X PBS. After that we determine the CFU in each sample by measurement of OD600 and the scaling equation OD 1.0 = 1.5×10^8 CFU/mL. Serial dilution using 1X PBS then provided a range of initial cell counts. We incubated each specific number of cells with various specific concentrations of LL-37 (F27W) in the test tube in PBS solution for 60 min at 30°C. After that we added 50 µL solution from each tube (each particular combination of cell number and peptide concentration) to polystyrene 96-well plates supplemented with 50 µL 2X EZRDM in each well. We measured the OD 595 ($OD_{t=0}$), incubated 6 h, and measured the OD 595 again ($OD_{t=6h}$). Thus we could determine the 6h OD increase ($\Delta OD = OD_{t=6h} - OD_{t=0}$) for each particular combination (Fig. S8). If the OD does not increase significantly after 6 h, then the initial LL-37 concentration was sufficient to kill that number of cells. For example, we found 20 µM LL-37 (F27W) can kill bacteria with initial count of 4.5×10^7 CFU/mL, but not a higher initial count. The ratio of total LL-37 copies to the initial

number of cells provides an upper bound on the number of copies absorbed per cell. The combination 20 μM LL-37 and 4.5 x 10^7 CFU/mL yields an upper bound of ~2.7 x 10^8 LL-37 absorbed per cell.

Next we measured the fraction of LL-37 copies bound to cells (Fig. S9) by measuring the concentration of unbound peptide left in the supernatant after incubation with the cells using HPLC and detection by absorbance at 280 nm (17). Specific combinations of peptide solutions were mixed with specific numbers of bacteria in PBS at 30°C for 60 min, as before. Cells were removed by centrifugation. Fractional binding of peptide was determined by measuring the peptide remaining in the supernatant using HPLC and detection by absorbance at 280 nm. The experimental supernatant was analyzed using an analytical C18 column of 4.6×100 mm. The elution gradient was reverse phase with pump A: 0.065% trifluoroacetic (TFA) in 100% water (v/v) and B: 0.05% TFA in 100% acetonitrile (v/v). Calibration was achieved by spiking each mixture with a known concentration of 20 μM of the free amino acid W. This provides an internal control for injection volume and detection sensitivity. The ratio of LL-37 (F27W) signal to free W signal was then compared to that of a peptide solution without cells, but otherwise treated identically.

For example, in the case of 20 μM LL-37, sterilization occurs up to 4.5 × 10^7 CFU/mL. At this cell number, we found the binding fraction was 79%. We calculate that there are no surviving CFUs when there are 2.1 × 10^8 peptides per cell as follows:

$$20\ \mu\text{M} \times 1\ \text{mL} = 2.0 \times 10^{-8}\ \text{mol} = 1.2 \times 10^{16}\ \text{total LL-37}$$

$$1.2 \times 10^{16}\ \text{total LL-37} / 4.5 \times 10^{7}\ \text{cells} = 2.7 \times 10^{8}\ \text{total LL-37/cell}$$

$$2.7 \times 10^{8}\ \text{total LL-37/cell} \times 0.79 = 2.1 \times 10^{8}\ \text{LL-37 absorbed/cell.}$$

Of course the conditions in these LL-37 uptake experiments are quite different from those in the microscopy experiments. The uptake experiments are carried out in PBS to avoid interference in the absorption measurements by the multitude of species present in EZRDM. The LL-37 concentration is 4 μM in the microscopy experiments and 20 μM in the uptake experiments. In addition, the incubation time of 60 min is substantially longer in the observation time window of the diffusion experiments. Also, in the microscopy experiments the flow of a constant concentration of LL-37 provides an unlimited source of peptide after membrane permeabilization has occurred. Nevertheless, we view ~10^8 LL-37 absorbed per cell as a sensible first estimate that enables us to think quantitatively about the diffusion effects.

Estimated binding constant of a +6 cationic peptide to DNA

To make plausible such strong electrostatic binding of LL-37 to DNA, we provide a rough estimate of the amount of LL-37 that will bind to DNA within the *E. coli* nucleoid once the membranes are permeabilized. Record and coworkers have carried out quantitative *in vitro* studies of the binding of polycationic peptides to ds-DNA (18). They write the binding equilibrium as: L + D ↔ LD, and the binding constant as K_{obs} = [LD]/[L]•[D]. Here L is the free peptide ligand of charge +Z, D represents a free (unblocked; see below) phosphate binding site along the DNA backbone, and LD represents the peptide bound to a phosphate site on DNA. K_{obs} is the phenomenological observed equilibrium constant in units of M^{-1}. It represents peptide binding strength in the limit of sparse binding density, *i.e.*, for one peptide molecule binding to otherwise empty DNA. In this model, each phosphate provides one binding site, although binding of the polypeptide to one site may block binding to other sites as the binding density increases.

Experimental binding data for $+Z$ peptides over a wide range of peptide charges and 1:1 salt concentrations $[M^+]$ fit the equation (18):

$$\log K_{obs} = A - 0.9\, Z \log [M^+].$$

The constant A is the value of $\log K_{obs}$ for $[M^+] = 1$ M. Our peptide uptake experiments were carried out in PBS solution, whose monovalent cation concentration is $[M^+] = 0.16$ M (primarily NaCl). For $Z = 6$ and $[M^+] = 0.16$ M, the equation yields $K_{obs} = 20{,}000\ M^{-1}$; the term A is negligible. Suppose there are N total phosphate binding sites within the nucleoid and that each peptide with $Z = +6$ occupies $n = 6$ adjacent phosphate sites, blocking them from additional binding. The McGhee–von Hippel model (19) provides an appropriate binding isotherm for this situation:

$$\frac{v}{x} = K_{obs}\,(1 - nv)\left(\frac{1 - nv}{1 - (n-1)v}\right)^{n-1}$$

Here ν is the equilibrium fractional occupancy of the N total binding sites and $x = [L]$ is the free ligand concentration.

In our growth conditions, the average cell contains ~2.2 chromosome equivalents $= 10.1 \times 10^6$ base pairs of DNA, providing $N = 2 \times 10^7$ individual phosphate binding sites. In the uptake experiments, after an initial LL-37 concentration of 20 μM was exposed to 4.5×10^7 CFU/mL, there remained 21% of the LL-37 concentration in the supernatant. Thus $x = 4.2$ μM. The parameters $n = 6$, $K_{obs} = 20{,}000\ M^{-1}$ and $x = 4.2$ μM then yield the estimate $\nu = 0.045$ in our uptake experiments. This is 27% of the maximum possible fractional occupancy, $\nu_{max} = 1/n = 0.166$, so the coverage is fairly dense. There are an estimated $\nu N \sim 9 \times 10^5$ bound LL-37 copies in the nucleoid. The nucleoid contains $\sim 1/\nu = 22$ phosphate charges per bound LL-37 copy.

We can also estimate the corresponding model concentration of bound LL-37 within the nucleoid region. In an earlier DNA imaging study under the same growth conditions (4), we estimated the volume occupied by the two (quite compact) nucleoid lobes to be ~0.1 μm^3. The 9 x 10^5 bound LL-37 within a 0.1 μm^3 nucleoid volume corresponds to a local LL-37 concentration of ~15 mM. This very rough estimate is similar to the experimentally estimated average LL-37 concentration bound within the entire cell, which was 90 mM.

Of course these estimates involve many approximations and assumptions. We are assuming that LL-37, which contains eleven positive and five negative charges, behaves as a +6 peptide. The McGhee-von Hippel model assumes random binding to a linear array of phosphate charges with ligands completely immobiled after binding. We further assume that the *in vitro* binding constant applies within the nucleoid, which is much more compact and crowded than solution-phase DNA. However, the calculation lends credence to the proposal that the large LL-37 uptake per cell arises from strong binding that is primarily electrostatic in nature.

Finally, we can write $K_{obs} = k_{on} / k_{off}$ to estimate how long an individual bound LL-37 copy remains bound before dissociating. If we assume the binding step is diffusion limited, then $k_{on} \sim 10^8 \ M^{-1}s^{-1}$. The estimate K_{obs} = 20,000 M^{-1} then yields $k_{off} \sim 5000 \ s^{-1}$, or $\tau_{bound} = 1/k_{off} \sim 200 \ \mu s$. This indicates that the electrostatic binding is quite transient. This estimate in turn suggests that much of the LL-37 remains bound within the cell during the 80-min rinsing period not because the LL-37 is bound irreversibly, but because the high density of binding sites sequesters LL-37 due to myriad binding, unbinding, and re-binding cycles.

SUPPLEMENTAL MOVIES

Movie S1. Tracking of DNA loci *Right2* for a total of 60 min at 12 s/frame. Exposure time 50 ms for each channel. Injection of 4 µM LL-37 begins at t = 0. *L eft:* Phase contrast. *Middle:* ParB-GFP fluorescence. *Right:* Sytox Orange.

Movie S2. Single-molecule movie of ribosomes (S2-mEos2) beginning 30 min after onset of treatment with 4 µM LL-37. Movie duration is 3 s; images acquired at 30 ms/frame. *Left:* Phase contrast. *Right:* S2-mEos2.

Movie S3. Single-molecule movie of HU-mEos2 beginning 30 min after onset of treatment with 4 µM LL-37. Movie duration is 3 s; images acquired at 30 ms/frame. *Left:* Phase contrast. *Right:* HU-mEos2.

Movie S4. Single-molecule movie of Kaede beginning 30 min after onset of treatment with 4 µM LL-37. Movie duration is 2 s; images acquired at 2 ms/frame. *Left:* Phase contrast. *Right:* Kaede.

Movie S5. Movies of the DNA locus *Right2* before and after treatment with 8 µM LL-37 (2X MIC) beginning at t = 0. Movie duration is 20 min, with images acquired at a rate of one frame per 12 s with exposure time of 50 ms. *Left:* Phase contrast. *Right:* ParB-GFP fluorescence. In some cells the loci remain punctal ; in others, loci seemingly dissolve, indicting the heterogeneity of LL-37 effects.

SUPPLEMENTAL FIGURES

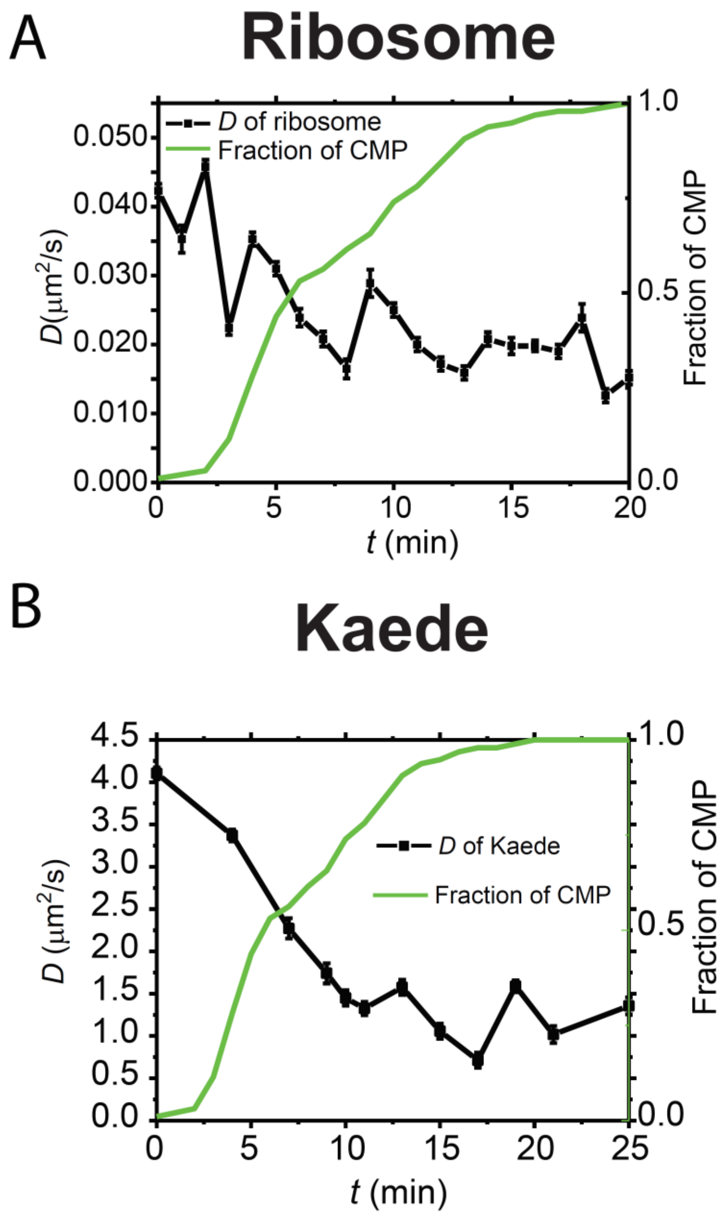

Figure S1. Mean diffusion coefficient of (A) ribosomes and (B) Kaede tetramer as a function of time after addition of 4 μM LL-37 (1X MIC) at $t = 0$. For ribosomes, each time point represents the average over a 30-s window with images taken at 30 ms/frame. For Kaede, each time point represents the average over a 20-s window with images taken at 2 ms/frame. In both panels, the green curve indicates the percentage of cells whose CM has been permeabilized (CMP), as judged by the onset of Sytox Orange fluorescence in a separate experiment.

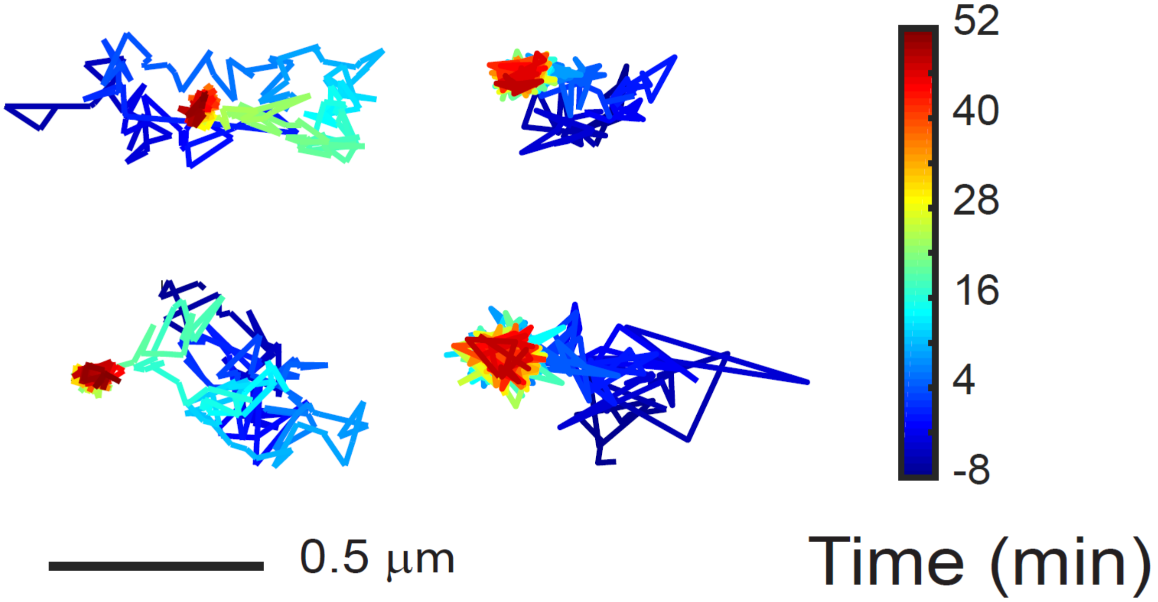

Figure S2. Further examples of 1-hr time lapse trajectories of the chromosome locus *Right2* obtained at 12 s/frame. Time in minutes is color-coded as shown, with t = 0 time of injection of 4 μM of LL-37 (1X MIC).

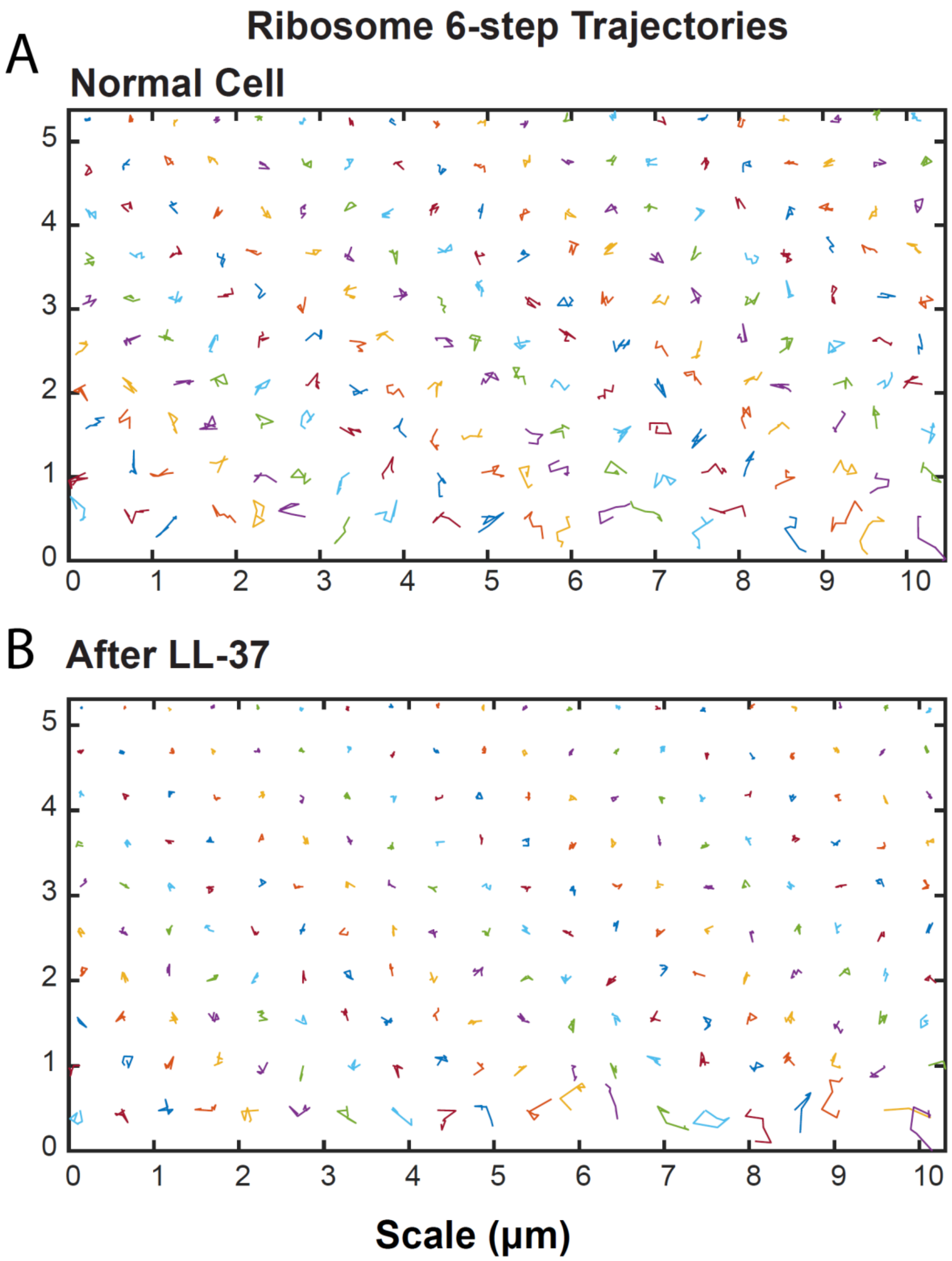

Figure S3. Galleries of 200 randomly selected 6-step trajectories of ribosomes (S2-mEos2 tracking) taken at 30 ms/frame in one field of view. (A) Cells in normal growth.
(B) LL-37-treated cells at $t > 15$ min after addition of 4 μM (1X MIC) LL-37. Scale is in μm. In each case, trajectories are arranged in order of increasing R_g. There are more compact trajectories and the most compact trajectories are smaller after LL-37 treatment.

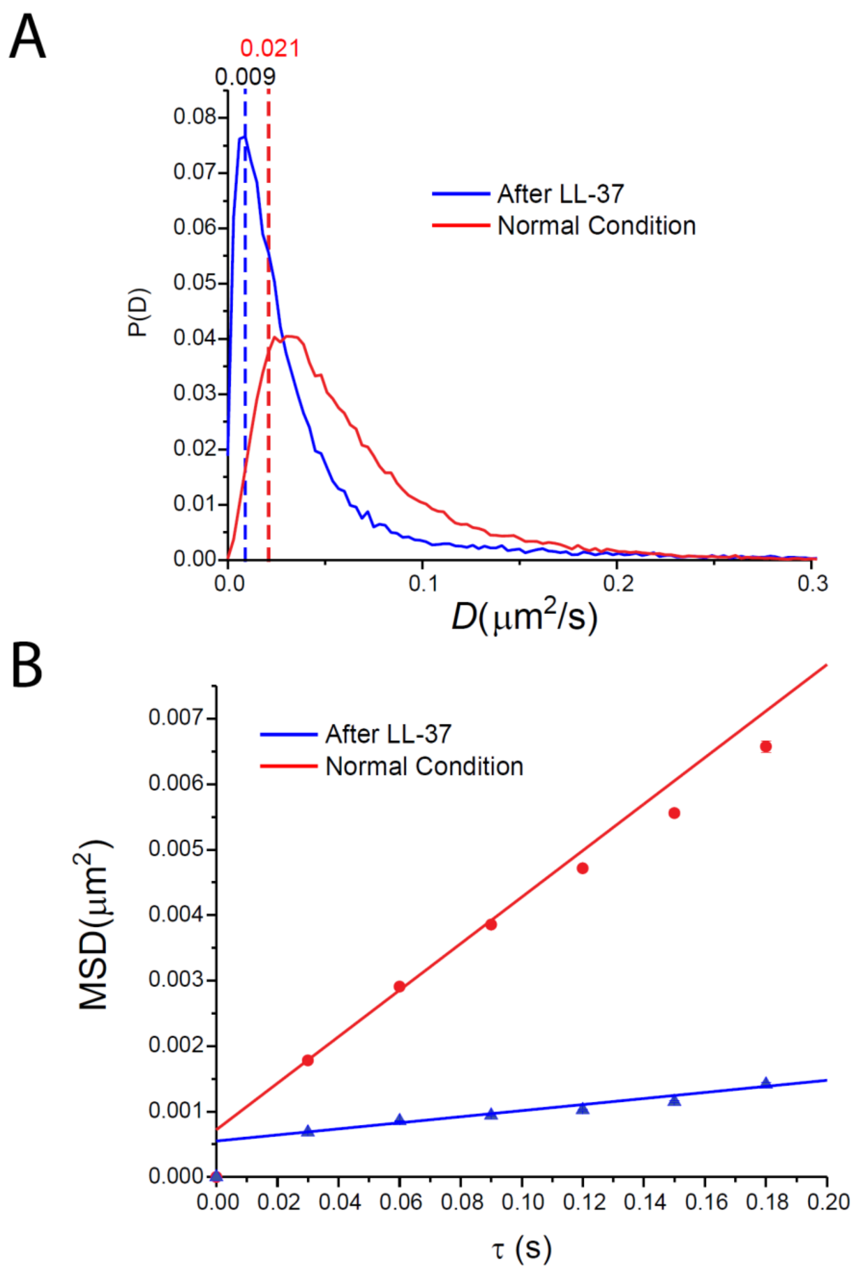

Figure S4. (A) Distributions of 6-step estimates of single-ribosome diffusion coefficients *D* for cells growing normally (red curve) and for cells at *t* > 15 min after addition of 4 μM of LL-37 (1X MIC, blue curve). Cutoffs for the slowest 15% in normal growth ($D < 0.021$ μm^2/s) and the slowest 15% after LL-37 treatment ($D < 0.009$ μm^2/s) are shown. (B) MSD plots for the slowest 15% of ribosome trajectories from (A). The intercepts yield dynamic localization error estimates σ_{slow} = 16 nm in normal growth and σ_{slow} = 12 nm after LL-37 treatment.

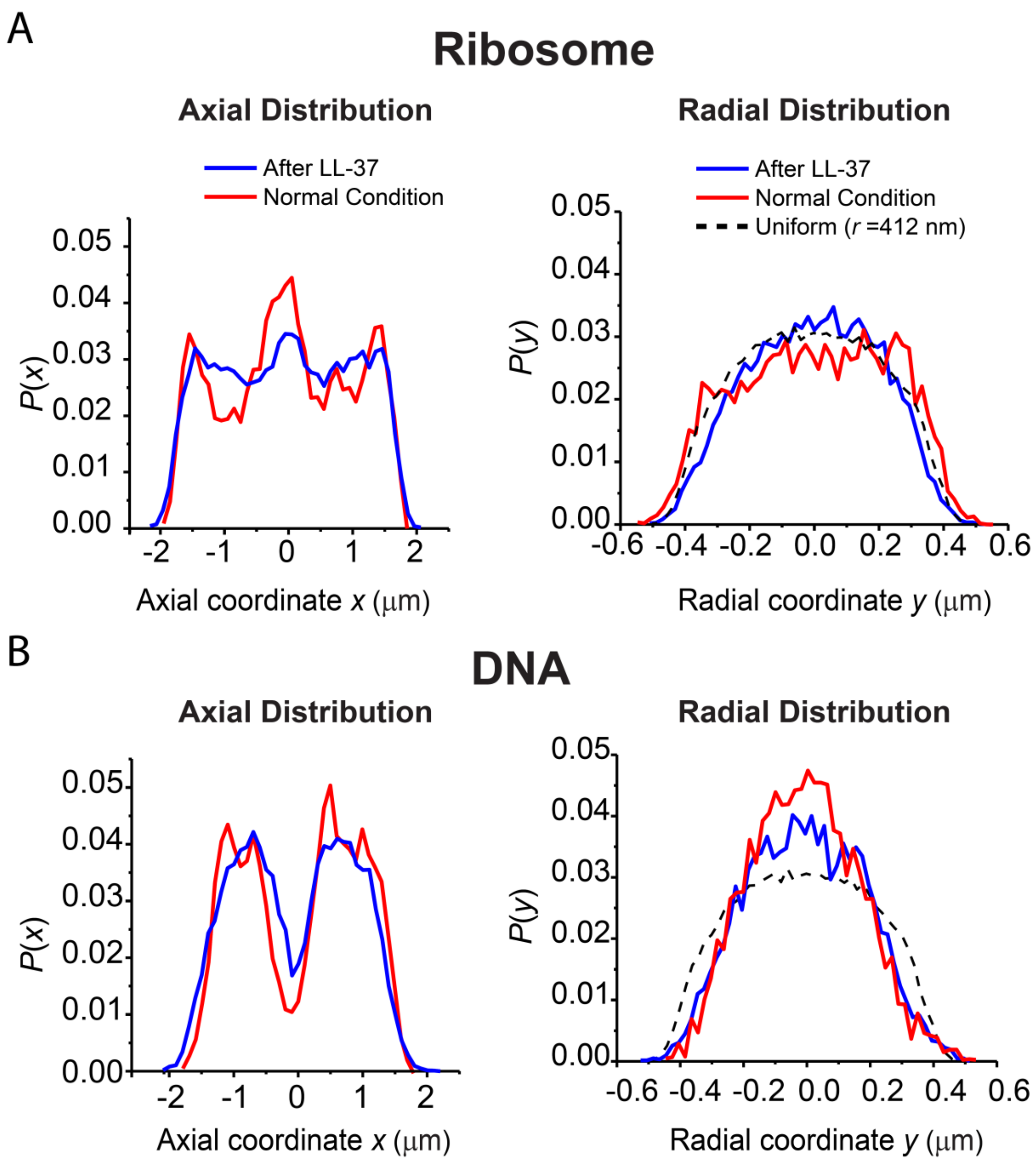

Figure S5. Ribosome and DNA axial and radial distributions compared for normal growth and after LL-37 treatment (1X MIC, $t > 15$ min). This is the same data used in Fig. 6 of the main text, re-plotted for facile comparison of the effects of LL-37 treatment on each distribution.

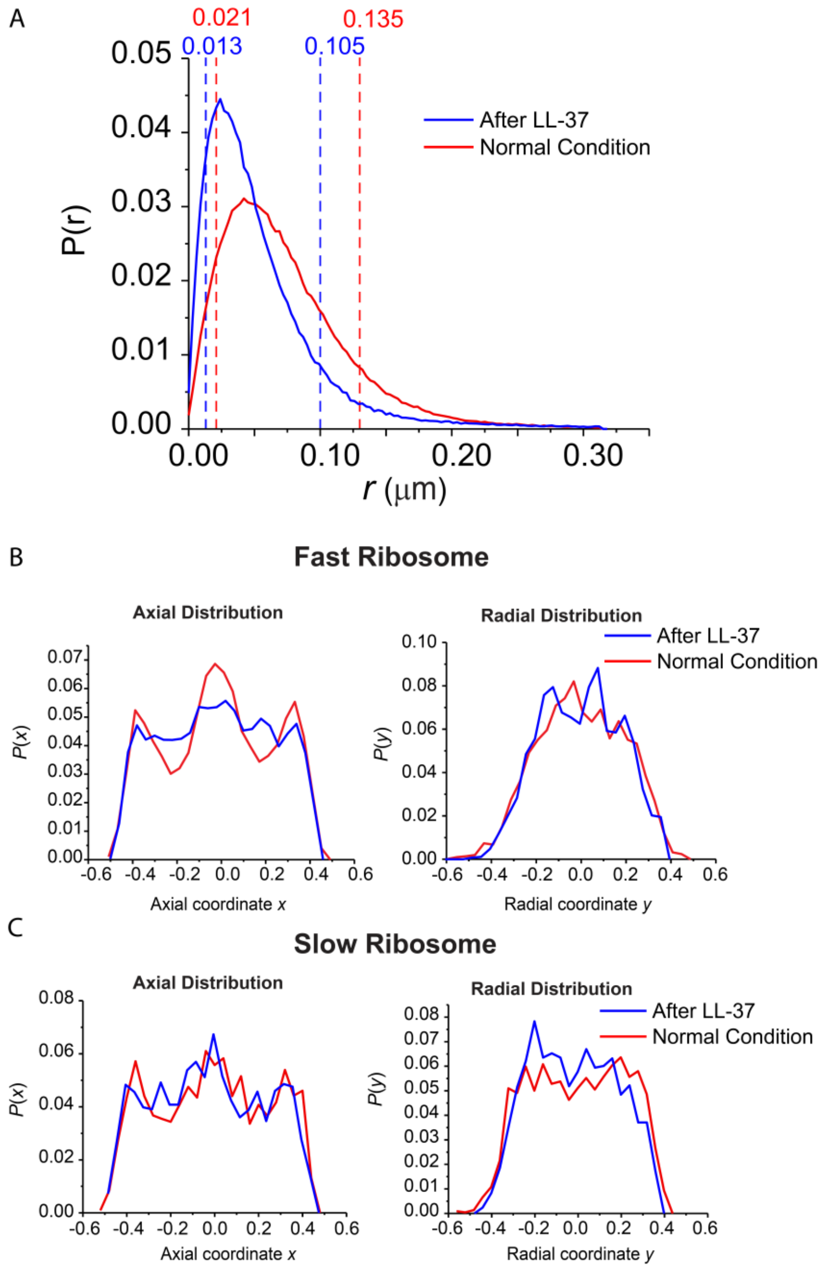

Figure S6. (A) Ribosome single-step distributions for normal cells and after LL-37 treatment as shown. In each case, dashed lines indicate cutoffs for the shortest 10% and the longest 10% of steps. These cutoffs were used to form axial and radial spatial distributions for slow and fast ribosomes before and after LL-37 treatment. (B) For cells in the length range 3.5-4.5 μm, fast ribosome axial and radial distributions for normal cells and for cells after LL-37 treatment. (C) For cells in the length range 3.5-4.5 μm, slow ribosome axial and radial distributions for normal cells and for cells after LL-37 treatment. Both coordinates are scaled by the cell length or diameter to lie in the range ± 0.5. Radial distribution includes only those localizations occurring in the nucleoid regions ($0.1 < |x| < 0.3$).

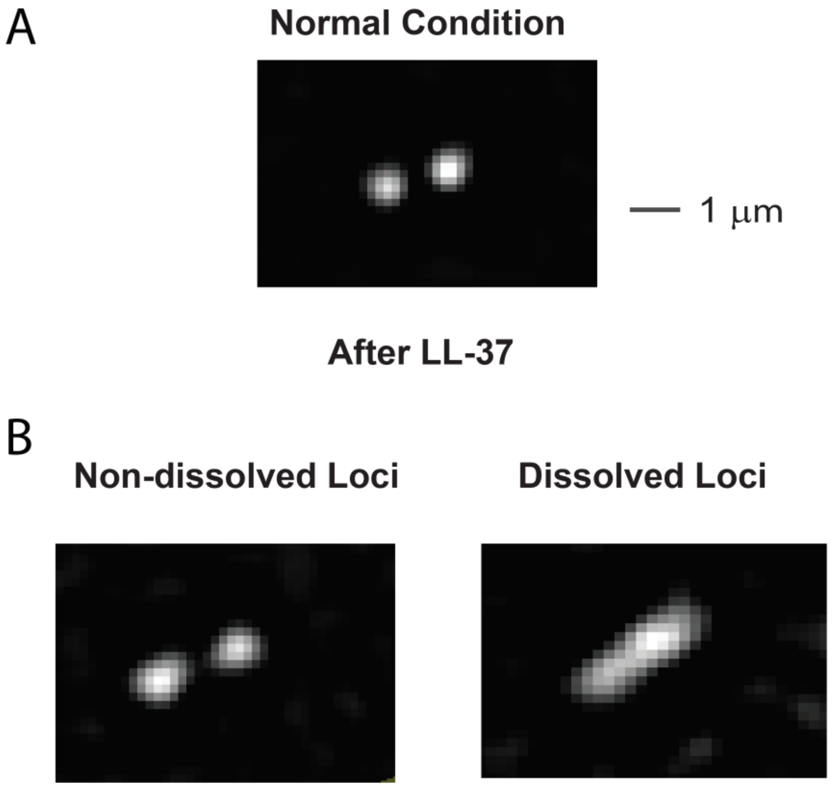

Figure S7. Images of the chromosomal locus *Right2*. (A) Normal growth. (B) After treatment with 8 μM LL-37 (2X MIC). In some cells loci remain punctal (left); in others, loci seemingly dissolve (right). (Dissolved loci, Non-dissolved loci)

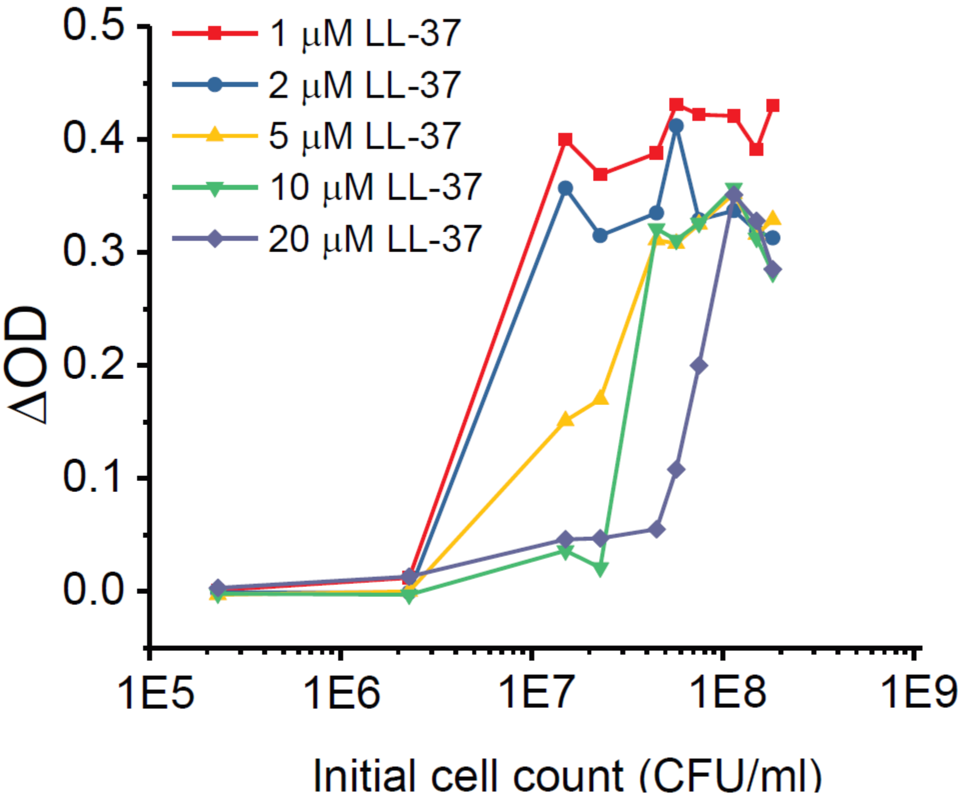

Figure S8. Determination of the LL-37 (F27W) initial concentration required to kill a given concentration of bacterial cells. For each LL-37 concentration and for each initial cell count, we measured the optical density at 595 nm before and after 6-hour incubation of the cells with LL-37. The change in OD is defined as $\Delta OD = OD_{t=6h} - OD_{t=0}$. For a given LL-37 concentration, as the CFU/ml increases, ΔOD becomes significantly greater than zero when the initial number of cells becomes too large to be completely killed by the LL-37 concentration provided. For the particular LL-37 concentration of 20 μM, this occurred at 4.5×10^7 CFU/ml.

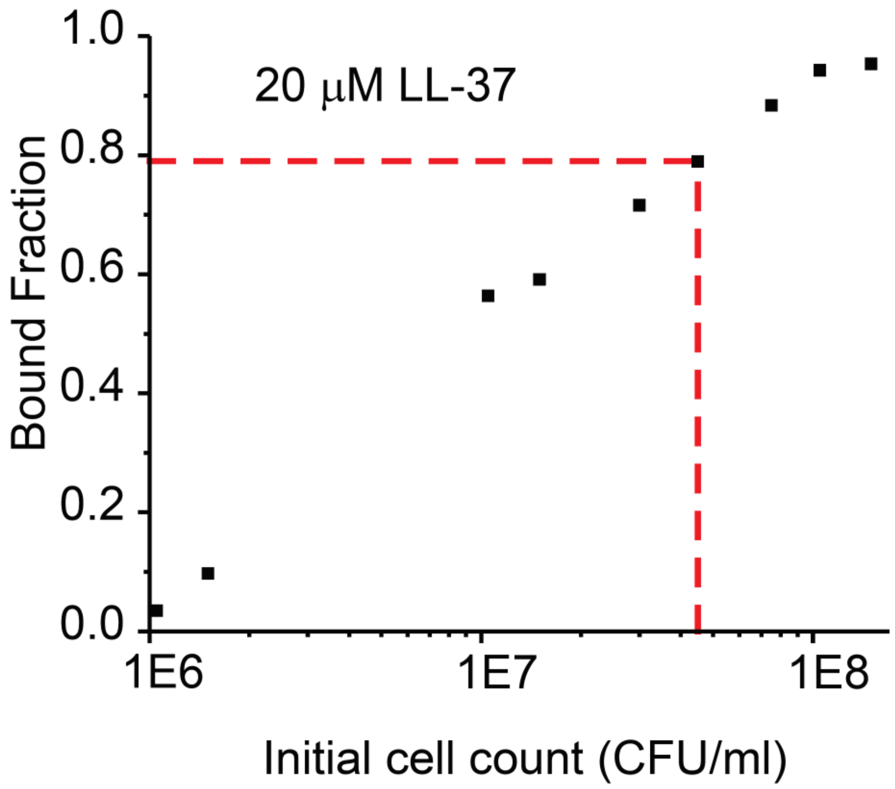

Figure S9. Fraction of peptide bound to cells vs initial cell count for LL-37 (F27W) concentration of 20 μM. Peptide was mixed at 20 μM with each concentration of bacterial cells in PBS at 30°C for 60 min, after which the cells were removed by centrifugation. Fractional binding of peptide was determined by measuring the peptide remaining in the supernatant using HPLC and absorbance at 280 nm and comparing it to a peptide solution without cells, but otherwise treated identically. Dashed lines indicate that 79% of the peptide was absorbed by 4.5×10^7 CFU/ml of cells, the largest number of cells that were completely killed at 20 μM of LL-37 (F27W), as determined from Fig. S8.

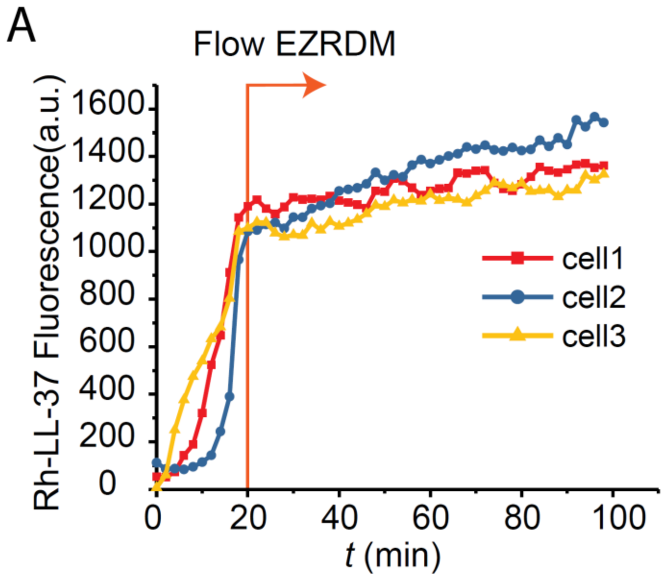

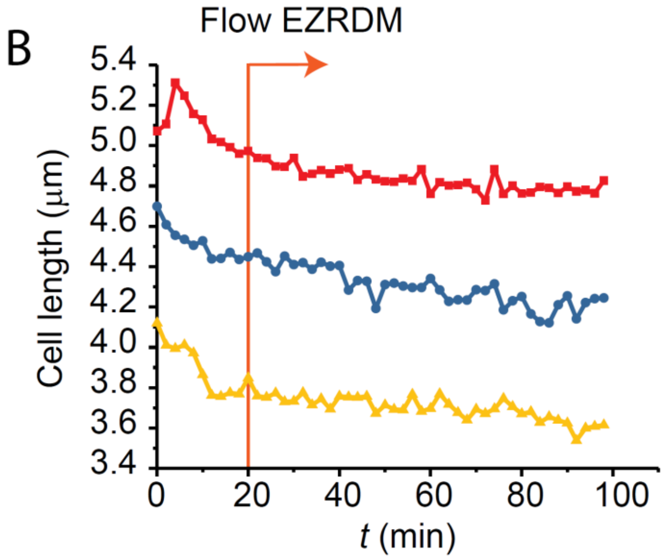

Figure S10. Recovery experiment. At t = 0-20 min, 4 μM Rh-LL-37 (1X MIC) was flowed across plated cells, enough time for membrane permeabilization of all cells. Over the interval t = 20-100 min, the flow was switched to fresh, aerated EZRDM in an attempt to wash away the intracellular Rh-LL-37 and restore growth. Throughout the 100-min observation time, phase contrast and fluorescence images were acquired every 2 min. (A) Three typical examples of Rh-LL-37 fluorescence intensity vs time. Rh-LL-37 remains bound within the cells. (B) For the same three cells, cell length vs time from phase contrast images. There is no evidence of recovery of growth.

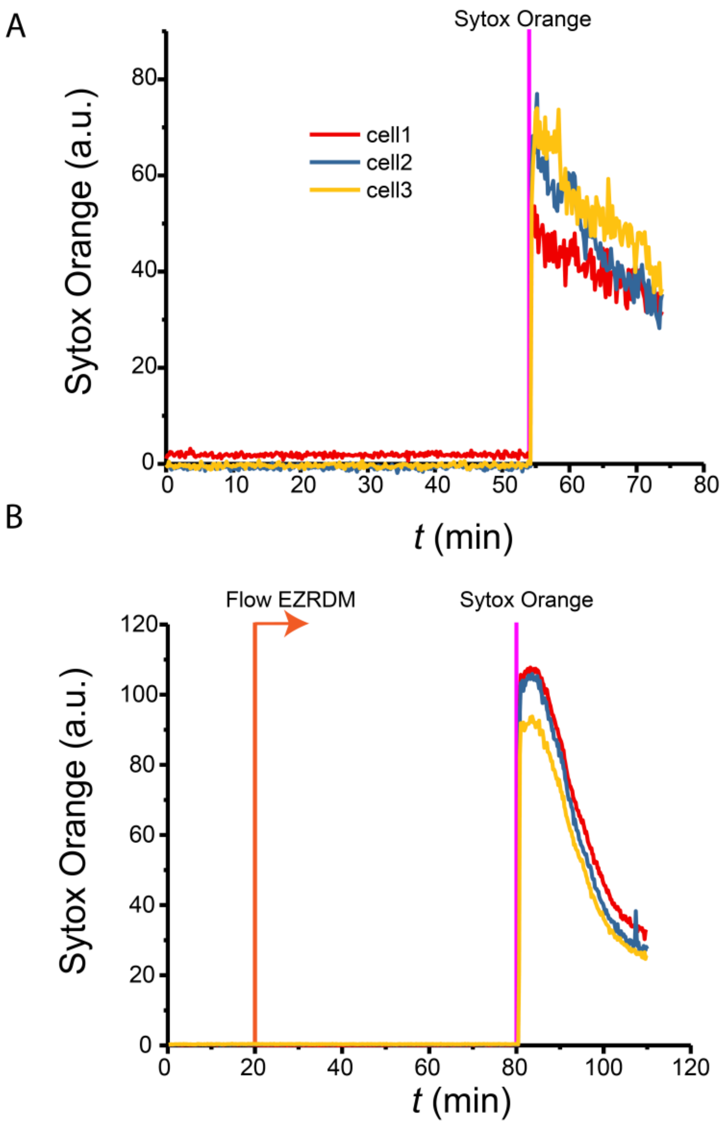

Figure S11. Tests for the persistence of membrane permeability after LL-37 attack. Throughout observation time, phase contrast and fluorescence images were acquired every 12 s. (A) 4 μM LL-37 (1X MIC) was flowed for 54 min and then the flow was switched to LL-37 with 5 nM Sytox Orange. An abrupt increase of Sytox Orange fluorescence intensity was observed in all cells within 12 s. Both membranes remain permeable to Sytox Orange for 60 min. (B) Over the interval t = 0-20 min, 4 μM LL-37 (1X MIC) was flowed across plated cells, sufficient time for membrane permeabilization of all cells. Over the subsequent interval t = 20-80 min, the flow was switched to fresh, aerated EZRDM, followed by rinsing with fresh medium plus Sytox Orange beginning at t = 80 min. Again the Sytox Orange fluorescence rises abruptly in all cells. Evidently both membranes remain permeable for 80 min after the initial attack by LL-37, even in the absence of LL-37 in the rinsing flow.

SUPPLEMENTAL REFERENCES

1. Espeli O, Mercier R, & Boccard F (2008) DNA dynamics vary according to macrodomain topography in the *E. coli* chromosome. *Mol Microbiol* 68(6):1418-1427.
2. Bakshi S, Dalrymple RM, Li W, Choi H, & Weisshaar JC (2013) Partitioning of RNA polymerase activity in live *Escherichia coli* from analysis of single-molecule diffusive trajectories. *Biophys J* 105(12):2676-2686.
3. Bakshi S, Choi H, Mondal J, & Weisshaar JC (2014) Time-dependent effects of transcription- and translation-halting drugs on the spatial distributions of the *Escherichia coli* chromosome and ribosomes. *Mol Microbiol* 94(4):871-887.
4. Mohapatra S & Weisshaar JC (2018) Functional mapping of the *E. coli* translational machinery using single-molecule tracking. *Mol Microbiol* 110(2):262-282.
5. Bakshi S, Bratton BP, & Weisshaar JC (2011) Subdiffraction-limit study of Kaede diffusion and spatial distribution in live *Escherichia coli*. *Biophys J* 101(10):2535-2544.
6. Bakshi S, Siryaporn A, Goulian M, & Weisshaar JC (2012) Superresolution imaging of ribosomes and RNA polymerase in live *Escherichia coli* cells. *Mol Microbiol* 85(1):21-38.
7. Choi H, Yang Z, & Weisshaar JC (2015) Single-cell, real-time detection of oxidative stress induced in *Escherichia coli* by the antimicrobial peptide CM15. *Proc Natl Acad Sci U S A* 112(3):E303-310.
8. Stannard JN & Horecker BL (1947) The *in vitro* Inhibition of Cytochrome Oxidase by Azide and Cyanide. *Fed Proc* 6(1):210-210.
9. Weber SC, Spakowitz AJ, & Theriot JA (2012) Nonthermal ATP-dependent fluctuations contribute to the *in vivo* motion of chromosomal loci. *Proc Natl Acad Sci U S A* 109(19):7338-7343.
10. Cavari BZ, Avidor Y, & Grossowi.N (1967) Effect of Carbonyl Cyanide m-Chlorophenylhydrazone on Respiration and Respiration-Dependent Phosphorylation in Escherichia Coli. *Biochem J* 103(2):601-&.
11. Weber SC, Spakowitz AJ, & Theriot JA (2010) Bacterial chromosomal loci move subdiffusively through a viscoelastic cytoplasm. *Phys Rev Lett* 104(23):238102.
12. Kralj JM, Hochbaum DR, Douglass AD, & Cohen AE (2011) Electrical Spiking in *Escherichia coli* Probed with a Fluorescent Voltage-Indicating Protein. *Science* 333(6040):345-348.
13. Crocker JC & Grier DG (1996) Methods of Digital Video Microscopy for Colloidal Studies. *Journal of Colloid and Interface Science* 179(1):298-310.
14. Sliusarenko O, Heinritz J, Emonet T, & Jacobs-Wagner C (2011) High-throughput, subpixel precision analysis of bacterial morphogenesis and intracellular spatio-temporal dynamics. *Molecular Microbiology* 80(3):612-627.
15. Michalet X (2010) Mean square displacement analysis of single-particle trajectories with localization error: Brownian motion in an isotropic medium. *Phys Rev E Stat Nonlin Soft Matter Phys* 82(4 Pt 1):041914.

16. Mohapatra S & Weisshaar J (2018) Modified Pearson Correlation Coefficient for Two-color Imaging in Spherocylindrical Cells. *bioRXiv*.

17. Starr CG, He J, & Wimley WC (2016) Host Cell Interactions Are a Significant Barrier to the Clinical Utility of Peptide Antibiotics. *ACS Chemical Biology* 11(12):3391-3399.

18. Record MT, Lohman TM, & Dehaseth P (1976) Ion Effects on Ligand-Nucleic Acid Interactions. *Journal of Molecular Biology* 107(2):145-158.

19. See, for example: Dill KA and Bromberg S in *Molecular Driving Forces*, 2nd Edition, Chapter 28, pp. 575-578 (Garland Science, London and New York, 2011).
